# Toluene oxidation with triple-module cooperativity in an atomically precise Cu_4_Pt_2_(C≡CCyOH)_8_ catalyst

**DOI:** 10.1093/nsr/nwag328

**Published:** 2026-05-29

**Authors:** Shisi Tang, Chenyang Shen, Qingxi Zhai, Yiqi Tian, Mingchen Han, Dan Luo, Gongde Wu, Xiuwen Wang, Weiping Ding, Yan Zhu

**Affiliations:** State Key Laboratory of Coordination Chemistry, Key Laboratory of Mesoscopic Chemistry of Ministry of Education, School of Chemistry and Chemical Engineering, Nanjing University, Nanjing 210023, China; State Key Laboratory of Coordination Chemistry, Key Laboratory of Mesoscopic Chemistry of Ministry of Education, School of Chemistry and Chemical Engineering, Nanjing University, Nanjing 210023, China; State Key Laboratory of Coordination Chemistry, Key Laboratory of Mesoscopic Chemistry of Ministry of Education, School of Chemistry and Chemical Engineering, Nanjing University, Nanjing 210023, China; State Key Laboratory of Coordination Chemistry, Key Laboratory of Mesoscopic Chemistry of Ministry of Education, School of Chemistry and Chemical Engineering, Nanjing University, Nanjing 210023, China; State Key Laboratory of Coordination Chemistry, Key Laboratory of Mesoscopic Chemistry of Ministry of Education, School of Chemistry and Chemical Engineering, Nanjing University, Nanjing 210023, China; State Key Laboratory of Coordination Chemistry, Key Laboratory of Mesoscopic Chemistry of Ministry of Education, School of Chemistry and Chemical Engineering, Nanjing University, Nanjing 210023, China; School of Environment Engineering, Nanjing Institute of Technology, Nanjing 211167, China; Center for Microscopy and Analysis, Nanjing University of Aeronautics and Astronautics, Nanjing 210016, China; State Key Laboratory of Coordination Chemistry, Key Laboratory of Mesoscopic Chemistry of Ministry of Education, School of Chemistry and Chemical Engineering, Nanjing University, Nanjing 210023, China; State Key Laboratory of Coordination Chemistry, Key Laboratory of Mesoscopic Chemistry of Ministry of Education, School of Chemistry and Chemical Engineering, Nanjing University, Nanjing 210023, China

**Keywords:** cluster, toluene oxidation, cooperativity, benzaldehyde, functional module

## Abstract

The dismantling and reassembly of C(*sp*^3^)-H bonds in hydrocarbons over both heterogeneous and homogeneous catalysts remain a fundamental challenge. Here, we report the toluene oxidation using an atomically precise Cu_4_Pt_2_(C≡CCyOH)_8_ cluster (HC≡CCyOH = 1-ethynyl-1-cyclohexanol) as the electrocatalyst with an exclusive selectivity for benzaldehyde accompanied by >99% Faradaic efficiency. Results reveal the systematic cooperativity of three functional modules combined into one cluster catalyst for six temporally subsequent events: the hydroxyl ligand of this cluster can capture water and then release it to the Cu sites, and water is sequentially activated on the Cu sites to form OH radicals; Pt sites engage in the abstraction of a hydrogen atom from the methyl group of toluene; the OH radicals then flip over to the Pt sites and react with dehydro-toluene to produce benzaldehyde, which is finally disentangled from the active sites with the help of the ligand. Meanwhile, the cluster is also used to catalyze the hydrogen evolution reaction at the cathode and exhibits much higher activity for hydrogen production than a typical Pt/C catalyst. Our study presents a molecular approach for designing highly active and selective catalysts for efficient inert molecule-involved reactions.

## Teaser

Toluene oxidation using an atomically precise Cu_4_Pt_2_(C≡CCyOH)_8_ cluster (HC≡CCyOH = 1-ethynyl-1-cyclohexanol) as the electrocatalyst is reported, with an exclusive selectivity for benzaldehyde accompanied by >99% Faradaic efficiency.

## INTRODUCTION

Selective hydrocarbon oxidation enables the transformation of readily available feedstocks into high-value oxygenated derivatives and thereby has attracted much attention in both academic research and industrial fields [1–3]. The process currently remains a challenge, primarily due to the activation and functionalization of C–H bonds as well as the unavoidable competition between desired partial oxidation and thermodynamically favored overoxidation [4]. A typical selective oxidation reaction of hydrocarbon is the toluene oxidation to benzaldehyde that is widely used in pharmaceuticals, agrochemicals, dye industries and fragrances [5]. The benzylic C–H bond of toluene is essentially non-polar and the bond dissociation energy is very high (88 kcal mol^−1^) [6]. Meanwhile, the product benzaldehyde is prone to be consecutively oxidized toward benzoic acid and eventually CO_2_. Therefore, it is highly desired to couple the increase in the selectivity of benzaldehyde with increased conversion of toluene. The industrial-scale synthesis of benzaldehyde traditionally relies on the chlorination and hydrolysis of toluene, which can give rise to the yield of benzaldehyde up to 97% [7]. However, this process is difficult to sustain, considering that it requires toxic chlorine gas and unavoidably generates the corrosive HCl. Efforts to relieve the above process by exploiting green strategies have been made, but little progress has been achieved toward this goal.

**Figure 1.**
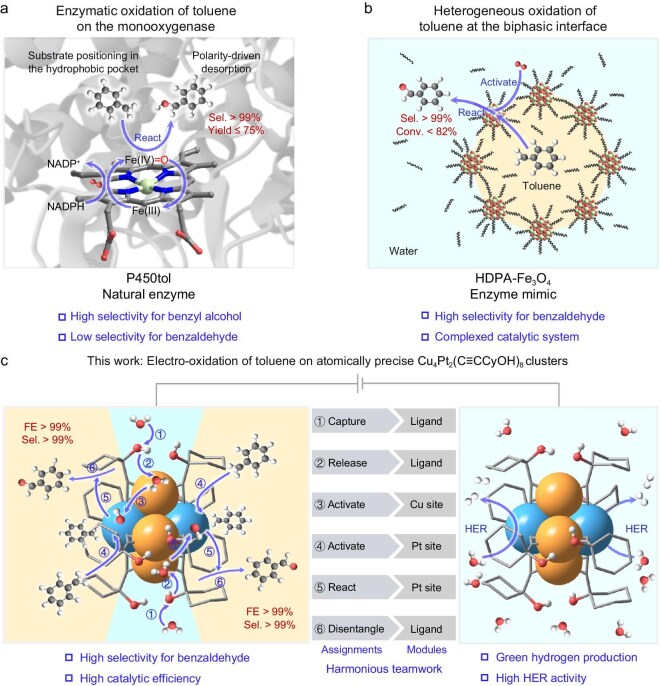
Strategies for selective oxidation of toluene. (a) Enzymatic catalysis of toluene oxidation over the monooxygenase P450tol. (b) Heterogeneous oxidation of toluene by the HDPA-Fe_3_O_4_ catalyst performed at the toluene/water interface. (c) Working principles of this study: selective oxidation of toluene to benzaldehyde over the Cu_4_Pt_2_(C≡CCyOH)_8_ cluster at the anode, accompanied by green hydrogen production over this cluster at the cathode. Harmonious teamwork: three modules of Cu sites, Pt sites and ligands orchestrated a catalytic cycle with six events from ① to ⑥ in turn. Color code: orange, Cu; light blue, Pt; green, Fe; red, O; dark blue, N; gray, C; white, H. H atoms bonded to carbon atoms from the ligands of Cu_4_Pt_2_(C≡CCyOH)_8_ were omitted for clarity.

Looking into catalysis of toluene oxidation in nature, as shown in Fig. 1a, the toluene monooxygenase P450tol is well known for its trilogy on converting toluene [8]: the methyl group of toluene is first oriented toward the metal site by a hydrophobic pocket, ensuring the oxidation occurring at the benzylic position; the redox partner NADPH (nicotinamide adenine dinucleotide phosphate) helps activate the oxygen, and thereby the metal site is invoked to activate the C–H bond of toluene; the polarity environment surrounding the active center can improve the desorption of the oxygenated product. Although the product is benzyl alcohol rather than benzaldehyde, the perfect coordination of the active sites and ambient medium in the natural enzyme could vivify our mental resources. We previously reported a hexadecylphosphate (HDPA)-functionalized iron oxide nanoparticle catalyst used in the toluene/water biphasic emulsion (Fig. 1b), where the catalytic center cooperated with the peripheral ligands [9,10]. The HDPA ligands bonded onto the Fe_3_O_4_ nanoparticles not only induced a hydrophobic surface on the active center, driving the access of the non-polar toluene to the Fe_3_O_4_ active center, but also promoted the exit of the polar benzaldehyde from the non-polar surface to the polar water phase, enabling highly selective oxidation of toluene to benzaldehyde. Nevertheless, the problem with this approach is the incomplete conversion that further pushes us to exploit a novel system.

Atomically precise metal cluster catalysts that can be crystallographically characterized are just like the protein structures in enzyme catalysis [11]. These cluster catalysts hold promise in controlling catalytic activity and selectivity by metal structure and surface ligands [12–14]. For example, Cu-based clusters capped by ligands have been reported to exhibit high catalytic performances in various chemical processes such as CO_2_ reduction, nitrate electroreduction and water electrooxidation [15–22]. Hence, we decided to challenge the toluene oxidation to benzaldehyde by designing atomically precise metal cluster catalysts. In this work, we successfully synthesized a hydroxy-functionalized alkyne ligand-protected Cu_4_Pt_2_(C≡CCyOH)_8_ cluster and achieved an unforeseen efficiency of toluene oxidation to benzaldehyde in the harmonious ecosystem. This cluster was comprised of three functional units such as Cu sites, Pt sites and ligands, in which each unit had its role to play while simultaneously working together as a good teamwork, as illustrated in Fig. 1c, undergoing the six distinct events from capturing, releasing, activation, reaction to disentangling in the sequence of the toluene oxidation process. In addition, the Pt sites of this cluster also exhibited a high activity for the hydrogen evolution reaction (HER), thereby realizing the dual production of benzaldehyde and green hydrogen in one integrated process. Our results highlighted the huge power of atomically precise cluster catalysts for achieving super catalytic performances for challenging chemical processes.

## RESULTS AND DISCUSSION

### Characterizations of Cu_4_Pt_2_(C≡CCyOH)_8_ cluster

**Figure 2.**
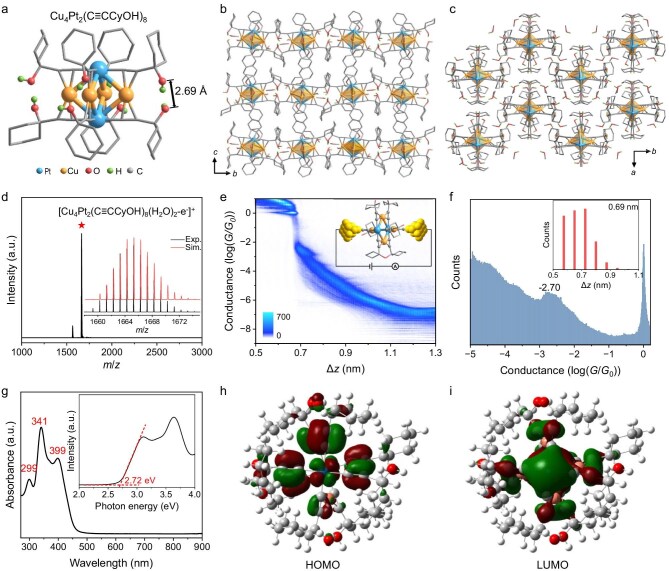
Structure analysis and physical properties of the Cu_4_Pt_2_(C≡CCyOH)_8_ cluster. (a) Crystal structure of Cu_4_Pt_2_(C≡CCyOH)_8_. (b) Packing pattern of Cu_4_Pt_2_(C≡CCyOH)_8_ viewed along the *a*-axis. (c) Packing pattern of Cu_4_Pt_2_(C≡CCyOH)_8_ viewed along the *c*-axis. 2D assembly was facilitated by methanol molecules as linear bridges. All H atoms bonded to carbon were omitted for clarity. (d) ESI-MS spectra of Cu_4_Pt_2_(C≡CCyOH)_8_. (e) 2D conductance-distance histogram of Cu_4_Pt_2_(C≡CCyOH)_8_. The inset shows the scheme of a single Cu_4_Pt_2_(C≡CCyOH)_8_ junction. (f) 1D conductance histogram of Cu_4_Pt_2_(C≡CCyOH)_8_ and the inset is the relative stretching distance distribution of Cu_4_Pt_2_(C≡CCyOH)_8_. (g) UV-vis spectra of Cu_4_Pt_2_(C≡CCyOH)_8_ and the inset was the corresponding photon-energy scale. (h, i) Frontier molecular orbitals of Cu_4_Pt_2_(C≡CCyOH)_8_.

The Cu_4_Pt_2_(C≡CCyOH)_8_ clusters were synthesized via a wet chemical method that was described in the supporting information. The crystal structure of Cu_4_Pt_2_(C≡CCyOH)_8_ is shown in Fig. 2a and its structural refinement is listed in Table S1. The cluster possessed a distorted octahedral Cu_4_Pt_2_ core, which was defined by a distorted Cu_4_ equatorial plane, with two platinum atoms occupying the trans-axial vertices. The Cu_4_Pt_2_ core was capped by eight 1-ethynyl-1-cyclohexanol (HC≡CCyOH) ligands, and each alkyne ligand adopted a μ_2_-η^1^, η^2^-bridging mode, forming a σ-bond to a Pt atom and simultaneously engaging in π-coordination to a Cu atom. The distances between Cu and Cu ranged from 3.214 to 3.390 Å (average: 3.301 Å) and the distance between Pt and Pt was 3.881 Å, indicating negligible Cu–Cu and Pt–Pt bonds. Conversely, the distances between Cu and Pt all fell between 2.957 and 3.105 Å (average: 3.035 Å), which were shorter than the sum of the van der Waals radii of Pt and Cu (∼3.1 Å), suggesting the presence of Cu–Pt bonds. The eight alkyne ligands were interconnected by four intracluster O–H···O hydrogen bonds between their hydroxyl groups. This internal hydrogen-bonding pattern resulted in O···O distances ranging from 2.690 to 2.869 Å (average: 2.742 Å), which enhanced the structural stability of Cu_4_Pt_2_(C≡CCyOH)_8_. Notably, the hydrogen bonding interactions between adjacent clusters in the crystal cell were also observed (Fig. S1) and they made the clusters arrange into an extensive two-dimensional (2D) supramolecular architecture (Fig. 2b and c). However, adjacent layers were connected by weak supramolecular interactions, specifically non-covalent H···H contacts (Fig. S2) [23].

Electrospray ionization-mass spectrometry (ESI-MS) further confirmed the formula of the cluster, as shown in Fig. 2d, where the main peak at *m/z* = 1665.3 was assigned to [Cu_4_Pt_2_(C≡CCyOH)_8_-e^−^]^+^ supported by the agreement between experimental and simulated isotopic patterns. The weak peak at *m/z* = 1565.4 was assigned to the [Cu_3_Pt_2_(C≡CCyOH)_8_–2e^−^]^+^ fragment formed from the ESI measurement (Fig. S3). From the analysis of X-ray photoelectron spectroscopy (XPS), as shown in Fig. S4, the Cu 2*p* XPS peaks were observed at 933.3 eV (2*p*_3/2_) and 953.3 eV (*2p*_1/2_), and Cu LMM (Auger transition) appeared at 914.8 eV with no shakeup satellite of Cu(II), indicating that the Cu species existed in the +1 valence state, while the Pt 4*f* XPS peaks at 73.8 eV (4*f*_7/2_) and 77.1 eV (4*f*_5/2_) might be assigned to Pt(II) [24].

Ultraviolet-visible (UV-vis) absorption spectrum of Cu_4_Pt_2_(C≡CCyOH)_8_ showed the three characteristic peaks at 299, 341 and 399 nm (Fig. 2g), respectively, corresponding to excitation energies of 4.15, 3.64 and 3.11 eV. Accordingly, the optical gap was estimated to be 2.72 eV based on the photon-energy scale spectrum (Fig. 2g inset). This value agreed reasonably well with the HOMO (highest occupied molecular orbital)—LUMO (lowest occupied molecular orbital) gap of 2.89 eV obtained from theoretical calculations (Fig. 2h and i). Seen from the Kohn–Sham orbital energy level diagram and the atomic orbital component in each orbit (Fig. S5), the metal atoms (Pt and Cu) mostly contributed to the population of the frontier orbital across all the levels, and the predominant peak at 341 nm was mainly attributed to the transitions of HOMO-9 → LUMO, HOMO-8 → LUMO and HOMO-1 → LUMO.

The charge transport property of a single Cu_4_Pt_2_(C≡CCyOH)_8_ cluster was investigated using the scanning tunneling microscope break junction technique. Analysis of the 2D conductance-distance traces and the corresponding one-dimensional (1D) conductance histogram (Fig. 2e and f) revealed most probable conductance of 10^−2.70^  *G*_0_. From the relative stretched distance distribution, the cluster length was evaluated to be 0.69 nm by accounting for the Au–Au snap back distance (Fig. 2f inset), which was very close to the crystal feature length of 0.66 nm between two diagonally opposed alkyne ligands of the cluster, suggesting that the electrons could easily transport through this entire cluster [25].

### Catalytic performance of Cu_4_Pt_2_(C≡CCyOH)_8_

**Figure 3.**
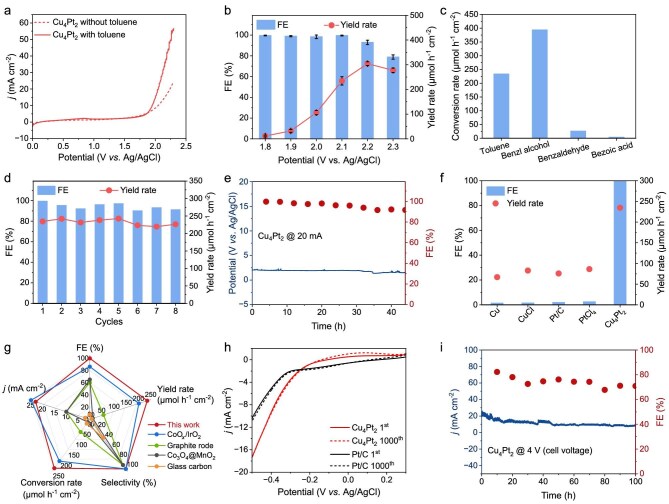
Electrocatalytic performances of Cu_4_Pt_2_ cluster catalysts for toluene oxidation to benzaldehyde and HER. (a) LSV curves for Cu_4_Pt_2_ recorded in acetone electrolyte containing 0.3 M TBAPF_6_, with and without 100 mM toluene. (b) Potential-dependent FEs and yield rates of benzaldehyde on the Cu_4_Pt_2_ catalyst. (c) Conversion rates of different reactants on Cu_4_Pt_2_. (d) Recyclability of Cu_4_Pt_2_ measured at 2.1 V versus Ag/AgCl. (e) Long-term stability test of Cu_4_Pt_2_ for toluene oxidation to benzaldehyde at a constant current density of 20 mA cm^−2^. (f) Comparison of FE and yield rate of benzaldehyde obtained from Cu powder, CuCl, Pt/C (20 wt.% Pt), PtCl_4_ and Cu_4_Pt_2_ under identical conditions at a potential of 2.1 V versus Ag/AgCl. (g) Comparison of current density, FE, yield rate and selectivity of benzaldehyde and toluene conversion rate of this work with previously reported catalysts. (h) LSV curves of Cu_4_Pt_2_ and 20 wt.% Pt/C in the solution of 0.1 M H_2_SO_4_ electrolyte. (i) Long-term stability of a two-electrode flow cell with Cu_4_Pt_2_ as both anode and cathode. Operating conditions: constant cell voltage = 4.0 V; electrolytes = 0.1 M H_2_SO_4_ (catholyte) and 0.3 M TBAPF_6_ in acetone with 100 mM toluene (anolyte); flow rate = 30 mL min^−1^.

We next explored the catalytic performance of Cu_4_Pt_2_(C≡CCyOH)_8_ (short for Cu_4_Pt_2_) cluster as the electrocatalyst for the selective oxidation of toluene to benzaldehyde. Figure 3a shows the linear scanning voltammetry (LSV) curves of Cu_4_Pt_2_ in electrolyte solutions with and without toluene. Upon the addition of toluene, an increase in current density was observed, suggesting that the cluster had catalytic activity for toluene oxidation. Furthermore, the current density–time (*I*–*t*) curves of Cu_4_Pt_2_ from 1.8 to 2.3 V (vs Ag/AgCl) were recorded (Fig. S6), and the products after *I*–*t* tests were analyzed by gas chromatography-mass spectrometry and ^1^H nuclear magnetic resonance spectroscopy (Figs S7 and S8). Figure 3b presents the Faradaic efficiency (FE) and yield rate of benzaldehyde from the toluene oxidation catalyzed by the Cu_4_Pt_2_ catalyst at various applied potentials. Over the potential range of 1.8–2.1 V (vs Ag/AgCl), the FEs for benzaldehyde remained consistently above 98%, while the yield rates increased progressively from 11.4 to 234.6 μmol h^−1^ cm^−2^. When the potential was increased to 2.2 V, the yield rate of benzaldehyde reached 304.3 μmol h^−1^ cm^−2^, accompanied by a slight decrease in FE due to the formation of a small amount of benzoic acid. A further increase to 2.3 V led to a pronounced drop in both FE and yield rate of benzaldehyde, likely due to the coupling and dehydration of the solvent acetone. Notably, benzyl alcohol was undetectable throughout the entire potential range, and benzoic acid was only generated in trace amounts at high potentials (Fig. S9).

Moreover, the control experiments were conducted at 2.1 V (vs Ag/AgCl) by, respectively, employing benzyl alcohol, benzaldehyde and benzoic acid as initial reactants. As shown in Fig. 3c, the conversion rate of benzyl alcohol reached 394.5 μmol h^−1^ cm^−2^, which was ∼1.7 times higher than that of toluene (234.6 μmol h^−1^ cm^−2^). In contrast, the conversion rates for benzaldehyde and benzoic acid were much lower, giving only 26.6 and 4.4 μmol h^−1^ cm^−2^, respectively. It revealed that benzyl alcohol was likely a reaction intermediate and then rapidly converted to benzaldehyde, while the subsequent oxidation of benzaldehyde to benzoic acid was significantly hindered.

Notably, the Cu_4_Pt_2_ catalyst showed a remarkable recyclability and maintained the high FE (>90%) and high yield rate (>210 μmol h^−1^ cm^−2^) for benzaldehyde (Fig. 3d). More importantly, the Cu_4_Pt_2_ clusters were highly robust during the reaction (Figs S10–S13). The chronoamperometry tests at 2.1 V versus Ag/AgCl for the duration ranging from 1 to 9 h revealed consistently high FE (>98% for benzaldehyde) and a cumulative benzaldehyde yield of 1990.5 μmol (Fig. S14). The chronopotentiometry test conducted at a constant current density of 20 mA cm^−2^ further showed that the system maintained a stable potential of ∼2.0 V versus Ag/AgCl for over 40 h with FE of benzaldehyde consistently above 90% (Fig. 3e).

To unveil the unique catalysis of Cu_4_Pt_2_ catalyst for toluene oxidation to benzaldehyde, we compared the catalytic properties of Cu- and Pt-based catalysts with Cu_4_Pt_2_ cluster. As shown in Fig. 3f, Cu powder, CuCl, 20 wt.% Pt/C and PtCl_4_ catalysts all exhibited negligible activities. Furthermore, the Cu_4_Pt_2_ catalyst was calcined at 200°C under an Ar flow to decompose the surface ligand, labeled as Cu_4_Pt_2_-Calc sample (Fig. S15). The calcined sample did not show the cluster aggregation, while carbon deposition was observed (Fig. S16). Compared to Cu_4_Pt_2_ protected by ligands, the Cu_4_Pt_2_-Calc sample exhibited a remarkable decrease in the FE for benzaldehyde to 70.2%, and the yield rate reduced to 218.1 μmol h^−1^ cm^−2^ (Fig. S17). The results indicated that both metal core and surface ligand of this cluster contributed to the overall catalytic performance and hence both were indispensable. We also systematically tested a series of metal clusters for toluene oxidation performances under identical conditions, including octahedral clusters such as Cu_6_(MPY)_6_ (MPY = 2-mercaptopyrimidine) [26], Pt_6_(PPh_3_)_4_Cl_5_ [27] and Cu_4_Pt_2_(C≡CPh)_8_ [28], as well as various homometallic Cu clusters. These clusters showed significantly low FEs and yield rates for benzaldehyde, as shown in Fig. S18. Such a comparison further indicated that the triple-module cooperativity in the Cu_4_Pt_2_(C≡CCyOH)_8_ cluster was indeed effective for the toluene oxidation to benzaldehyde, in which the Cu sites, Pt sites and ligands simultaneously worked together as a good teamwork to improve the oxidation process. Additionally, the Cu_4_Pt_2_ cluster catalyst benchmarked superior performance to other reported catalysts for toluene oxidation to benzaldehyde [29–32] (Fig. 3g and Table S2).

Motivated by the requirement to pair anodic toluene oxidation with an efficient HER cathode, we also investigated the HER performance on the Cu_4_Pt_2_ catalyst. As shown in Fig. 3h, at a current density of 10 mA cm^−2^, Cu_4_Pt_2_ exhibited a lower overpotential (−0.41 V vs Ag/AgCl) compared to 20 wt.% Pt/C (−0.49 V vs Ag/AgCl), demonstrating the excellent HER catalytic activity of the Cu_4_Pt_2_. Furthermore, the long-term stability of Cu_4_Pt_2_ for the HER at a fixed overpotential of −0.41 V versus Ag/AgCl was achieved, as shown in the current density–time curve (Fig. S19).

To evaluate the practical application of the Cu_4_Pt_2_ catalyst for the toluene oxidation concurrent with HER, we assessed its stability in a two-electrode flow cell configuration, employing Cu_4_Pt_2_ as both cathode and anode catalysts. In this setup, a higher cell voltage of 4.0 V was required to achieve a comparable current density of ∼20 mA cm^−2^ (Fig. 3i), which was attributed to a substantial liquid junction potential. The durability of this system was evaluated by chronopotentiometry, which indicated the stable performance over 100 h under a constant applied voltage. Throughout this test, the FEs for benzaldehyde always remained above 65%, while a gentle decrease in the current density was correlated with the depletion of toluene concentration in the anolyte. The application potentiality of the Cu_4_Pt_2_ catalyst was of utmost importance in order to put it to large-scale use of such catalyst.

### Mechanistic study

**Figure 4.**
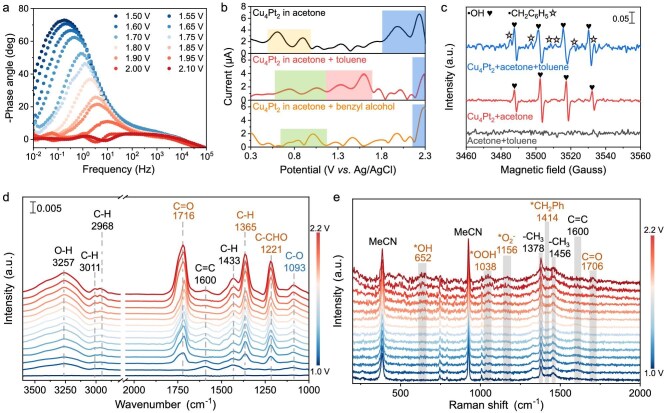
Mechanistic investigation of the toluene oxidation over the Cu_4_Pt_2_ catalyst. (a) The potential-dependent Bode phase angle plots over Cu_4_Pt_2_. (b) The fifth harmonic FTACV curves of Cu_4_Pt_2_ recorded in acetone electrolyte (0.3 M TBAPF_6_) without a substrate, and in the presence of toluene or benzaldehyde. (c) EPR spectra of Cu_4_Pt_2_ measured at 1.8 V versus Ag/AgCl after 10 min in acetone electrolyte (0.3 M TBAPF_6_) with DMPO as the spin trap, with and without toluene. (d) *In situ* ATR-FTIR spectra of the Cu_4_Pt_2_ system recorded in acetone electrolyte (0.3 M TBAPF_6_) with toluene, at applied potentials from 1.0 to 2.2 V versus Ag/AgCl. (e) *In situ* Raman spectra of Cu_4_Pt_2_ system recorded in acetonitrile electrolyte (0.3 M TBAPF_6_) with toluene, at applied potentials from 1.0 to 2.2 V versus Ag/AgCl.

We then turned our attention to the mechanistic investigation to unravel the origin of excellent performances of Cu_4_Pt_2_. To explore the interfacial dynamics of the catalyst, we employed *in situ* electrochemical impedance spectroscopy to probe the interfacial charge-transfer processes [33]. Figure S20 displays the Nyquist plots of Cu_4_Pt_2_ measured at different potentials in the reaction electrolyte. Within the potential range of 1.5–2.1 V (vs Ag/AgCl), the diameter of the Nyquist semicircles for the catalyst decreased with increasing potential, indicating accelerated charge-transfer kinetics for the toluene oxidation reaction, which aligned with the prediction of the Butler–Volmer equation. From the potential-dependent Bode plots (Fig. 4a), with the potential increase, the transition was observed from a single peak to two distinct peaks, revealing that the toluene oxidation system shifted toward a mechanism involving both charge-transfer process and diffusion process at high potentials. The inflection point in the low-frequency region was observed at 1.7 V (vs Ag/AgCl), suggesting the significant occurrence of toluene oxidation reaction. The inflection points in the Bode plot progressively shifted toward higher frequencies with increasing potentials, implying that the reaction underwent fast charge-transfer kinetics. Concurrently, the decrease in phase angles reflected a transition from charge transfer toward diffusion-limited control for the Cu_4_Pt_2_ reaction system.

Moreover, the electron-transfer characteristics of the catalyst during toluene oxidation were investigated using Fourier-transformed alternating current voltammetry (FTACV). Given that the higher-order harmonic components of FTACV minimize the interference from non-Faradaic processes and provide enhanced sensitivity to underlying Faradaic signatures [34], we specifically analyzed the fifth harmonic component to probe the electron-transfer behavior. In the acetone electrolyte containing a small amount of water (Fig. 4b, upper panel), Cu_4_Pt_2_ exhibited pronounced current responses at ∼0.70 V and 2.05 V (vs Ag/AgCl), which were assigned to the oxygen evolution reaction (OER) from water (yellow region) and side reactions involving acetone (blue region), respectively. Upon the introduction of toluene (Fig. 4b, middle panel), two new current peaks emerged at ∼1.45 and 0.90 V (vs Ag/AgCl), which was tentatively assigned to the toluene oxidation to benzyl alcohol (pink region) and the benzyl alcohol oxidation to benzaldehyde (green region), respectively. Concurrently, the OER current peak disappeared, while the acetone-related current peak shifted to ∼2.30 V (vs Ag/AgCl). When only benzyl alcohol was added to the acetone electrolyte, the current peaks were exclusively observed in the green and blue regions (Fig. 4b, lower panel). These above observations showed that the adsorption of toluene onto the catalyst surface effectively suppressed the side reactions. In addition, it was observed that the open-circuit potential decreased on the Cu_4_Pt_2_ upon toluene injection (Fig. S21), further validating the affinity of toluene adsorbed onto the cluster.

To probe the reactive intermediates generated in the Cu_4_Pt_2_ catalytic system, *in situ* electron paramagnetic resonance (EPR) measurements using 5,5-dimethyl-1-pyrroline *N*-oxide (DMPO) as a spin-trapping agent were conducted. Under a constant applied potential in acetone electrolyte and in the presence of Cu_4_Pt_2_, the characteristic EPR signal of hydroxyl radicals (•OH) was detected, reflecting one-electron oxidation of water (red curve in Fig. 4c). Upon the addition of toluene to the electrolyte, characteristic EPR signals of both •OH and benzyl radicals (•CH_2_C_6_H_5_) were detected [35,36] (blue curve in Fig. 4c; Fig. S22). Subsequently, a series of quenching experiments were conducted to further elucidate the essential roles of these intermediates in the Cu_4_Pt_2_-catalyzed toluene oxidation. As shown in Fig. S23, the addition of *tert*-butanol (TBA, •OH scavenger) and butylated hydroxytoluene (BHT, carbon-centered radical scavenger) suppressed the benzaldehyde yield rate by 72.8% and 49.1%, respectively. While quenching of superoxide radicals (•O_2_^−^) with benzoquinone (superoxide radical scavenger) also led to a reduction in benzaldehyde production, the extent of inhibition was less pronounced compared to that observed with TBA and BHT. These results indicated that both •OH and •CH_2_C_6_H_5_ served as pivotal reactive species in this reaction. Given the critical role of •OH radicals derived from water, we investigated the influence of water content on the FEs of benzaldehyde in the toluene oxidation processes. Figure S24 shows that the trace water in ordinary acetone was optimal, while anhydrous acetone or excess water might lead to the decrease of benzaldehyde FEs, implying that an appropriate amount of water in the reaction was crucial for optimizing reaction conditions.

*In situ* attenuated total reflection Fourier transform infrared spectroscopy (ATR-FTIR) was used to further monitor the potential-dependent intermediate species in toluene oxidation. Figure 4d displays the infrared (IR) signals collected within the 3600–1000 cm^−1^ range under applied potentials from 1.0 to 2.2 V (vs Ag/AgCl) over Cu_4_Pt_2_. As the reaction started, the IR bands of the C–H stretching vibration of aromatic ring (∼3011 cm^−1^), the C–H stretching vibrations of −CH_2_^−^ (∼2968 cm^−1^), the symmetric C=C stretching vibrations of aromatic ring (∼1600 cm^−1^) and the in-plane C–H bending vibrations of −CH_3_ (1433 cm^−1^) from toluene were observed [37]. The appearance of these bands accompanied by gradual enhancement in intensity implied the adsorption of toluene on Cu_4_Pt_2_. The band at 1093 cm^−1^ was attributed to the C–O stretching vibration of benzyl alcohol and the band at 3257 cm^−1^ was assigned to the O–H stretching vibration [38], suggesting the generation of benzyl alcohol and the adsorption of water on the catalyst. As the applied potential increased, the characteristic bands of benzaldehyde appeared and gradually strengthened: the band at 1716 cm^−1^ was assigned to C=O stretching vibration, the band at 1365 cm^−1^ was attributed to C–H deformation vibration of aldehyde group and the band at 1221 cm^−1^ was related to C–CHO stretching vibrations [10,39,40]. These observations indicated that Cu_4_Pt_2_ catalyzed the toluene oxidation by first adsorbing and activating toluene and water to form benzyl alcohol and subsequently benzyl alcohol being oxidized to benzaldehyde. It was noted that benzoic acid was not detected throughout the whole process, implying the stability of benzaldehyde against further oxidation, which accounted for the high selectivity for benzaldehyde obtained in this cluster system.

*In situ* Raman spectra experiments were conducted to further study the reaction species generated in the reaction catalyzed by Cu_4_Pt_2_ (Fig. 4e). The bands at 385 and 925 cm^−1^ were attributed to the solvent (acetonitrile), and the bands at 1378 and 1456 cm^−1^ were assigned to the −CH_3_ group of toluene. The band at 1414 cm^−1^ was assigned to the methylene −CH_2_ deformation vibration of *CH_2_C_6_H_5_, indicating the dehydrogenation of the methyl group of toluene [41,42]. The vibration band of *OH (652 cm^−1^) revealed the adsorption and dissociation of water molecules on the surface of Cu_4_Pt_2_, which was the key oxygen species of toluene oxidation. Despite the progressive intensification of the bands for *OOH (1038 cm^−1^) and *O_2_^−^ (1156 cm^−1^), these radicals were largely quenched and hence contributed little to toluene oxidation [43,44]. And with the increase of potential, C=O stretching vibration appeared at 1706 cm^−1^, showing the generation of benzaldehyde in this system [45]. Overall, after the deprotonation of toluene on the catalyst to form *CH_2_C_6_H_5_, it rapidly coupled with *OH and was subsequently oxidized to benzaldehyde.

*In situ* electrochemical Raman spectroscopy was also employed to monitor H_2_ evolution process in 0.1 M H_2_SO_4_ electrolyte over Cu_4_Pt_2_. As shown in Fig. S25, the bands at 1332 and 1596 cm^−1^ were respectively assigned to the *D* and *G* bands of carbon materials. The band at 2178 cm^−1^ was assigned to the Pt–H stretching vibration [46], which initially emerged and intensified in the low negative potential region, indicating the adsorption and accumulation of H atoms on Pt sites of the cluster. Upon further increasing the negative potential, the Pt–H band intensity declined, likely due to the rapid formation and detachment of H_2_ at high reaction rates.

**Figure 5.**
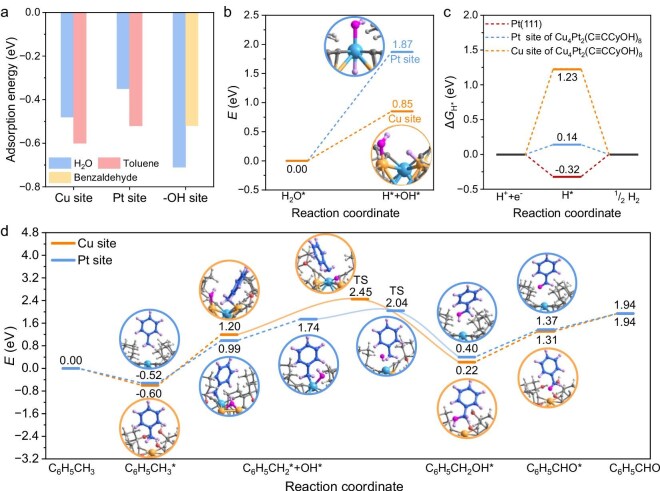
DFT calculations. (a) The adsorption of toluene and water on different sites of Cu_4_Pt_2_(C≡CCyOH)_8_, and the adsorption of benzaldehyde on the −OH site from the ligand of the cluster. (b) Comparison of the H_2_O dissociation between Cu site and Pt site of Cu_4_Pt_2_(C≡CCyOH)_8_. (c) Calculated adsorption free energies of H* on different sites of Cu_4_Pt_2_(C≡CCyOH)_8_, with the Pt (111) slab as a reference. (d) Energy profiles for toluene oxidation to benzaldehyde over different sites of Cu_4_Pt_2_(C≡CCyOH)_8_. Insets show the corresponding intermediates involved in each elementary step. Color code: orange, Cu; light blue, Pt; red, O from ligands; gray, C from ligands; white, H from ligands; magenta, O from reactants; dark blue, C from reactants; lavender, H from reactants.

Density functional theory (DFT) calculations were carried out to provide in-depth insight into the underlying reaction mechanism. The optimized configuration of Cu_4_Pt_2_(C≡CCyOH)_8_ is shown in Fig. S26. We tested different sites for the adsorption of toluene and water and the results are summarized in Fig. 5a. As for the adsorption of H_2_O, it can be clearly seen that the Cu site of Cu_4_Pt_2_(C≡CCyOH)_8_ exhibited a higher adsorption energy (−0.48 eV) than the Pt site (−0.35 eV). The adsorption of toluene also followed a similar trend. Moreover, the −OH groups from the ligands possessed much stronger affinity for H_2_O molecules, indicating a potential for substantial accumulation of water molecules in the vicinity of the ligands. Meanwhile, a comparison of H_2_O dissociation on different sites of the cluster revealed that this process was thermodynamically more favorable over the Cu site (Fig. 5b). Therefore, it was reckoned that H_2_O was initially captured by the −OH groups of the cluster and subsequently transferred to the Cu sites, where it underwent dissociation to provide the oxygen source for the oxidation process. As shown in Fig. 5c, compared to the Cu site (1.23 eV), the Pt site exhibited a more optimal value of 0.14 eV for boosting the H* combination step. More importantly, it should also be noted that the H* adsorption-free energy on the Pt site was closer to zero than that on the Pt(111) slab, demonstrating the advantage of this cluster for H* adsorption and H_2_ generation.

Energy profiles of toluene oxidation to benzaldehyde over Cu_4_Pt_2_(C≡CCyOH)_8_ were displayed in Fig. 5d. Although the first elementary step, namely, toluene adsorption was a little bit advantageous over the Cu site (*E*_ads_ = −0.60 eV) than Pt site (*E*_ads_ = −0.52 eV), the barrier for subsequent oxidative dehydrogenation of activated toluene to C_6_H_5_CH_2_* over the Pt site was 0.29 eV lower. Since Cu was the main site for generating OH*, the reaction process of C_6_H_5_CH_2_* and OH* to form C_6_H_5_CH_2_OH* on the Cu site required an energy barrier of 1.25 eV. However, as for the Pt site, the OH* on Cu (Cu-OH) may migrate to the Pt surface (Pt-OH), which was an endothermal step with a reaction energy of +0.75 eV. At this point, the reaction barrier for OH* to combine with the adsorbed C_6_H_5_CH_2_* was only 0.3 eV. More than that, the oxidation of C_6_H_5_CH_2_OH* to C_6_H_5_CHO* was also more favorable on the Pt site. The desorption of C_6_H_5_CHO* over both sites were basically the same. The notable adsorption affinity of the −OH site for C_6_H_5_CHO (*E*_ads_ = −0.52 eV) also contributed to the desorption process (Fig. 5a), thereby suppressing further oxidation of benzaldehyde. Overall, these theoretical findings further suggested the concerted catalysis within this cluster for the toluene-to-benzaldehyde conversion.

## CONCLUSION

In summary, we have successfully synthesized an atomically precise Cu_4_Pt_2_(C≡CCyOH)_8_ cluster and found its exclusive capability for the selective oxidation of toluene toward benzaldehyde. Based on the molecular devise, three functional units are combined into this cluster catalyst and show an unprecedented teamwork, which can capture and activate H_2_O, oxidize toluene and clean the product from the cluster, respectively, thereby giving rise to almost 100% selectivity for benzaldehyde with >99% FE. Meanwhile this cluster can also be applied to produce green hydrogen at the cathode via the HER progress. The efficient co-production of benzaldehyde and H_2_ indeed underscores the practical potential of this cluster system. This Cu_4_Pt_2_(C≡CCyOH)_8_ example with the abundant collaborations among surface ligand and metal core, metal and metal in the core and surface ligand and surface metal, has demonstrated the specificity of atomically precise cluster catalysts for challenging intractable chemical processes. We anticipate that these atomically precise catalysts will ultimately bring catalysis science to a new level.

## Supplementary Material

nwag328_Supplemental_Files
