# Supplementary material for: Toluene oxidation with triple-module cooperativity in an atomically precise Cu4Pt2(C≡CCyOH)8 catalyst
Source: Natl Sci Rev. 2026 May 29;13(13):nwag328. doi: 10.1093/nsr/nwag328 (PMC13373964; doi:10.1093/nsr/nwag328)

checkCIF/PLATON report

Structure factors have been supplied for datablock(s) 1

THIS REPORT IS FOR GUIDANCE ONLY. IF USED AS PART OF A REVIEW PROCEDURE FOR PUBLICATION, IT SHOULD NOT REPLACE THE EXPERTISE OF AN EXPERIENCED CRYSTALLOGRAPHIC REFEREE.

No syntax errors found.   CIF dictionary   Interpreting this report

Datablock: 1

Bond precision:	C-C = 0.0105 A		Wavelength=0.71073
Cell:	a=13.9992(5)	b=21.3655(8)	c=23.4954(8)
	alpha=90	beta=101.678(1)	gamma=90
Temperature:	193 K		

	Calculated	Reported
Volume	6882.0(4)	6882.0(4)
Space group	P 21/c	P 1 21/c 1
Hall group	-P 2ybc	-P 2ybc
Moiety formula	C64 H88 Cu4 O8 Pt2, 3(C H4 O)	C64 H88 Cu4 O8 Pt2, 3(C H4 O)
Sum formula	C67 H100 Cu4 O11 Pt2	C67 H100 Cu4 O11 Pt2
Mr	1725.84	1725.80
Dx,g cm-3	1.666	1.666
Z	4	4
Mu (mm-1)	5.319	5.319
F000	3448.0	3448.0
F000'	3441.13	
h,k,lmax	18,27,30	18,27,30
Nref	16003	15876
Tmin,Tmax	0.593,0.587	0.617,0.746
Tmin'	0.582	

Correction method= # Reported T Limits: Tmin=0.617 Tmax=0.746
AbsCorr = NONE

Data completeness= 0.992   Theta(max)= 27.617

R(reflections)= 0.0461(12578)   wR2(reflections)= 0.0952(15876)

S = 1.056   Npar= 772

The following ALERTS were generated. Each ALERT has the format
test-name_ALERT_alert-type_alert-level.
Click on the hyperlinks for more details of the test.

Alert level C

```
PLAT031_ALERT_4_C Refined Extinction Parameter Within Range of ...      3.308 Sigma
PLAT220_ALERT_2_C NonSolvent   Resd 1  C   Ueq(max)/Ueq(min) Range        3.1 Ratio
PLAT342_ALERT_3_C Low Bond Precision on  C-C Bonds ..............    0.01047 Ang.
PLAT906_ALERT_3_C Large K Value in the Analysis of Variance ......      2.474 Check
PLAT911_ALERT_3_C Missing FCF Refl Between Thmin & STh/L=    0.600          7 Report
                2  0  0,   0  2  0,   9  3  0,   0  4  0,  -2  0  2,  -3  1  2,
               14  0  8,
PLAT977_ALERT_2_C Check Negative Difference Density on H00B      .      -0.36 eA-3
PLAT977_ALERT_2_C Check Negative Difference Density on H6        .      -0.36 eA-3
PLAT977_ALERT_2_C Check Negative Difference Density on Hd        .      -0.36 eA-3
PLAT977_ALERT_2_C Check Negative Difference Density on H02O      .      -0.44 eA-3
PLAT977_ALERT_2_C Check Negative Difference Density on H02P      .      -0.37 eA-3
```

Alert level G

```
PLAT003_ALERT_2_G Number of Uiso or U(i,j)  Restrained non-H-Atoms         66 Report
PLAT007_ALERT_5_G Number of Unrefined Donor-H Atoms ..............         11 Report
              H007  H008  H009  H00A  H00B  H00C  H00J  H00M  H00W  H010  H011
PLAT083_ALERT_2_G SHELXL Second Parameter in WGHT  Unusually Large      50.89 Why ?
PLAT178_ALERT_4_G The CIF-Embedded .res File Contains SIMU Records          1 Report
PLAT188_ALERT_3_G A Non-default SIMU Restraint Value has been used     0.0100 Report
PLAT343_ALERT_2_G Unusual sp?      Angle Range in Main Residue for       C00D Check
PLAT343_ALERT_2_G Unusual sp?      Angle Range in Main Residue for       C00E Check
PLAT343_ALERT_2_G Unusual sp?      Angle Range in Main Residue for       C00F Check
PLAT343_ALERT_2_G Unusual sp?      Angle Range in Main Residue for       C00G Check
PLAT343_ALERT_2_G Unusual sp?      Angle Range in Main Residue for       C00H Check
PLAT343_ALERT_2_G Unusual sp?      Angle Range in Main Residue for       C00I Check
PLAT343_ALERT_2_G Unusual sp?      Angle Range in Main Residue for       C00K Check
PLAT343_ALERT_2_G Unusual sp?      Angle Range in Main Residue for       C00L Check
PLAT343_ALERT_2_G Unusual sp?      Angle Range in Main Residue for       C00P Check
PLAT343_ALERT_2_G Unusual sp?      Angle Range in Main Residue for       C00T Check
PLAT343_ALERT_2_G Unusual sp?      Angle Range in Main Residue for       C00U Check
PLAT343_ALERT_2_G Unusual sp?      Angle Range in Main Residue for       C00V Check
PLAT367_ALERT_2_G Long?  C(sp?)-C(sp?) Bond  C00T     - C00Z     .       1.50 Ang.
PLAT720_ALERT_4_G Number of Unusual/Non-Standard Labels ..........        165 Note
              Pt01    Pt02    Cu03    Cu04    Cu05    Cu06    O007    H007
              O008    H008    O009    H009    O00A    H00A    O00B    H00B
              O00C    H00C    C00D    C00E    C00F    C00G    C00H    C00I
              O00J    H00J    C00K    C00L    O00M    H00M    C00N    C00O
              C00P    C00Q    C00R    C00S    C00T    C00U    C00V    O00W
              H00W    C00X    C00Y    C00Z    O010    H010    O011    H011
              C012    C013    H01A    H01B    C014    C015    H01C    H01D
              C016    C017    H01E    H01F    C018    C019    H01G    H01H
              C01A    H01I    H01J    C01B    H01K    H01L    C01C    H01M
              H01N    C01D    H01O    H01P    C01E    H01Q    H01R    C01F
              H01S    H01T    C01G    H01U    H01V    C01H    H01W    H01X
              C01I    H01Y    C01J    H01Z    Ha      C01K    H01     Hb
              C01L    Hc      C01M    Hd      C01N    He      C01O    Hf
              C01P    Hg      C01Q    Hh      C01R    Hi      C01S    Hj
              C01T    Hk      C01U    Hl      C01V    Hm      C01W    Hn
```

```
              C01X    Ho      C01Y    Hp      C01Z    Hq      C020    H02A
              H02B    C021    H02S    H02T    H02U    C022    H02C    H02D
              C023    H02E    H02F    C024    H02G    H02H    C026    H02I
              H02J    C027    H02K    H02L    C028    H02M    H02N    C029
              H02Y    Hr      Hs      C02A    H02O    H02P    C02B    H02V
              H02W    H02X    C02D    H02Q    H02R
PLAT860_ALERT_3_G Number of Least-Squares Restraints ............       1398 Note
PLAT883_ALERT_1_G Absent Datum for _atom_sites_solution_primary ..     Please Do !
PLAT910_ALERT_3_G Missing FCF Reflection(s) Below Theta(Min)[Deg]=       1.83 Note
                1  0  0,   1  1  0,   0  1  1,   0  0  2,
PLAT912_ALERT_4_G Missing # of FCF Reflections Above STh/L=  0.600        116 Note
PLAT913_ALERT_3_G Missing # of Very Strong Reflections in FCF ....          2 Note
                0  4  0,  -3  1  2,
PLAT941_ALERT_3_G Average HKL Measurement Multiplicity ..........         3.9 Low
PLAT969_ALERT_5_G The 'Henn et al.' R-Factor-gap value ..........       1.618 Note
              Predicted wR2: Based on SigI**2  5.89 or SHELX Weight  9.02
PLAT978_ALERT_2_G Number C-C Bonds with Positive Residual Density.          4 Info
```

---

   0 **ALERT level A** = Most likely a serious problem - resolve or explain
   0 **ALERT level B** = A potentially serious problem, consider carefully
  10 **ALERT level C** = Check. Ensure it is not caused by an omission or oversight
  27 **ALERT level G** = General information/check it is not something unexpected

   1 ALERT type 1 CIF construction/syntax error, inconsistent or missing data
  22 ALERT type 2 Indicator that the structure model may be wrong or deficient
   8 ALERT type 3 Indicator that the structure quality may be low
   4 ALERT type 4 Improvement, methodology, query or suggestion
   2 ALERT type 5 Informative message, check

It is advisable to attempt to resolve as many as possible of the alerts in all categories. Often the minor alerts point to easily fixed oversights, errors and omissions in your CIF or refinement strategy, so attention to these fine details can be worthwhile. In order to resolve some of the more serious problems it may be necessary to carry out additional measurements or structure refinements. However, the purpose of your study may justify the reported deviations and the more serious of these should normally be commented upon in the discussion or experimental section of a paper or in the "special_details" fields of the CIF. checkCIF was carefully designed to identify outliers and unusual parameters, but every test has its limitations and alerts that are not important in a particular case may appear. Conversely, the absence of alerts does not guarantee there are no aspects of the results needing attention. It is up to the individual to critically assess their own results and, if necessary, seek expert advice.

Publication of your CIF in IUCr journals

A basic structural check has been run on your CIF. These basic checks will be run on all CIFs submitted for publication in IUCr journals (*Acta Crystallographica*, *Journal of Applied Crystallography*, *Journal of Synchrotron Radiation*); however, if you intend to submit to *Acta Crystallographica Section C* or *E* or *IUCrData*, you should make sure that full publication checks are run on the final version of your CIF prior to submission.

Publication of your CIF in other journals

Please refer to the *Notes for Authors* of the relevant journal for any special instructions relating to CIF submission.

PLATON version of 04/06/2025; check.def file version of 30/05/2025

Datablock 1 - ellipsoid plot

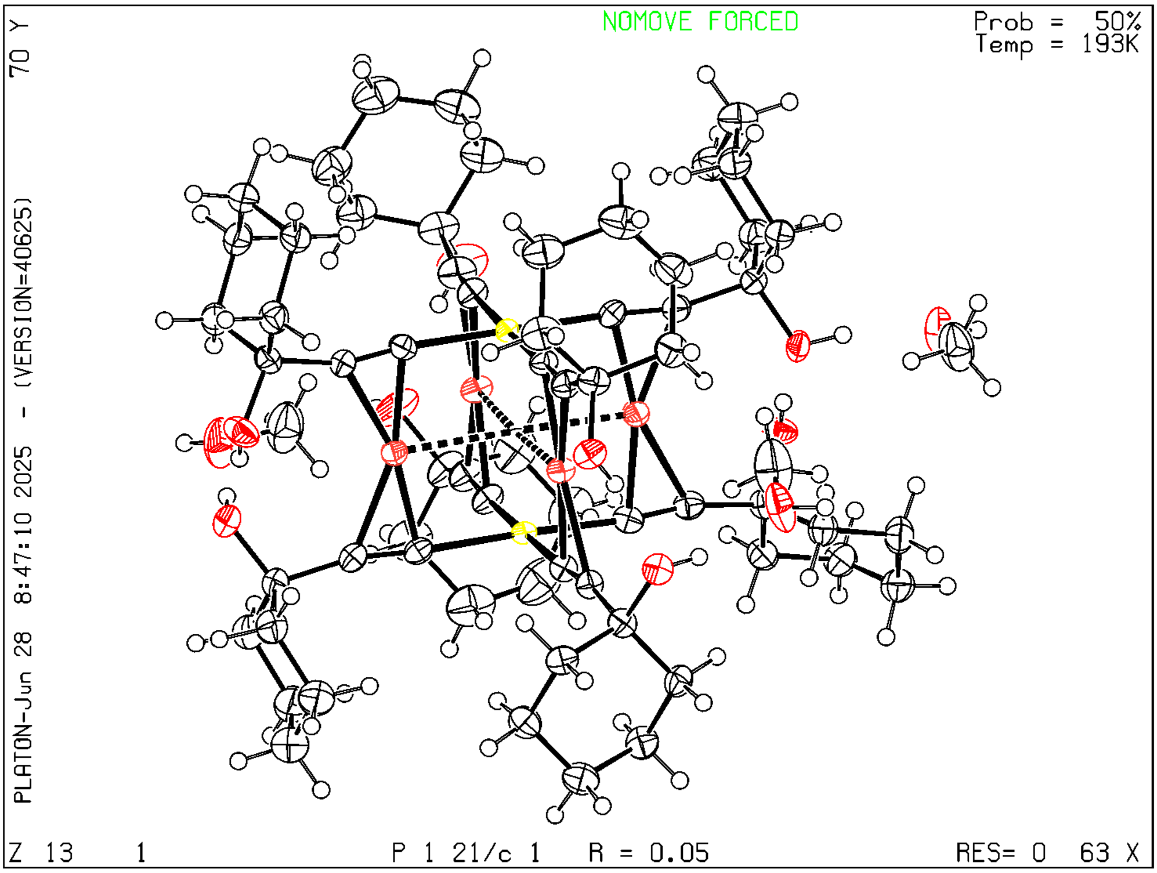

Supplement: nwag328_Supplemental_Files [file nwag328_supplemental_files.zip › checkcif_Cu4Pt2.pdf]
