# Supplementary material for: Toluene oxidation with triple-module cooperativity in an atomically precise Cu4Pt2(C≡CCyOH)8 catalyst
Source: Natl Sci Rev. 2026 May 29;13(13):nwag328. doi: 10.1093/nsr/nwag328 (PMC13373964; doi:10.1093/nsr/nwag328)
Supplement: nwag328_Supplemental_Files [file nwag328_supplemental_files.zip › SI-clean.pdf]

Supporting Information

Toluene oxidation with triple-module cooperativity in an atomically precise $Cu_4Pt_2(C{\equiv}CCyOH)_8$ catalyst

Shisi Tang[1,†], Chenyang Shen[1,†], Qingxi Zhai[1], Yiqi Tian[1], Mingchen Han[1], Dan Luo[1], Gongde Wu[2], Xiuwen Wang[3], Weiping Ding[1], and Yan Zhu[1,*]

[1]State Key Laboratory of Coordination Chemistry, Key Laboratory of Mesoscopic Chemistry of Ministry of Education, School of Chemistry and Chemical Engineering, Nanjing University, Nanjing 210023, China.

[2]School of Environment Engineering, Nanjing Institute of Technology, Nanjing 211167, China.

[3]Center for Microscopy and Analysis, Nanjing University of Aeronautics and Astronautics, Nanjing 210016, China.

***Corresponding author.** E-mail: zhuyan@nju.edu.cn

[†]Equally contributed to this work.

1. Experimental Section

Synthesis of $Cu_4Pt_2(C{\equiv}CCyOH)_8$ clusters

37.5 mg $Cu(NO_3)_2$ and 10.0 mg $[Pt(cod)Cl_2]_2$ were added into 5 mL of a mixed acetonitrile-methanol solvent (3:2, v/v). After complete dissolution, 24 mg 1-ethynyl-1-cyclohexanol (HC≡CCyOH) was added and the mixture was stirred for 5 min. Then, 200 μL triethylamine was added in one portion, causing the solution to change from light blue clear solution to a grass-green turbid suspension. After stirring for another 20 min, 50 μL of an ethanolic $NaBH_4$ solution (10 mg mL^{-1}) was introduced, turning the mixture dark green. The reaction was stirred at 60 °C for 12 h, then allowed to stand and cool slowly to room temperature, yielding orange-red cubic crystals.

Synthesis of $Cu_6(MPY)_6$ clusters

$Cu_6(MPY)_6$ (MPY = 2-mercaptopyrimidine) was synthesized based on the reported literature [S1]. 37.2 mg $Cu(CH_3CN)_4PF_6$ and 11.2 mg MPY were added into 20 mL of a mixed acetonitrile-methanol solvent (1:1, v/v). The reaction was stirred at room temperature for 5 min, and the yellow precipitate was collected by centrifugation to give the $Cu_6(MPY)_6$ clusters.

Synthesis of $Pt_6(PPh_3)_4Cl_5$ clusters

$Pt_6(PPh_3)_4Cl_5$ was synthesized based on the reported literature [S2]. 400 μL of 50 mM PPh_3 in toluene and 300 μL of 50 mM $H_2PtCl_6{\cdot}6H_2O$ in water were added to 9 mL ethanol under stirring. Then, 200 μL of 100 mM borane-tert-butylamine complex in ethanol was added. After 3 h of reaction, the solvent was removed and the residue was extracted with dichloromethane to give the brown $Pt_6(PPh_3)_4Cl_5$ clusters.

Synthesis of $Cu_4Pt_2(C{\equiv}CPh)_8$ clusters

$Cu_4Pt_2(C{\equiv}CPh)_8$ was synthesized based on the reported literature [S3]. 37.5 mg $Cu(NO_3)_2$, 37.0 mg $[Pt(cod)Cl_2]_2$ and 50 μL HC≡CPh were added into 10 mL acetone. After complete dissolution, 200 μL of triethylamine was added, yielding a pale yellow

clear solution. After stirring for 20 min, 200 μL of an ethanolic $NaBH_4$ solution (10 mg mL^{-1}) was introduced. After 3 h of reaction, the desired cluster was isolated by thin-layer chromatography.

Synthesis of $[CuPPh_3Cl]_4$ clusters

$[CuPPh_3Cl]_4$ was synthesized based on the reported literature [S4]. 49.5 mg CuCl and 1.31 g PPh_3 were added into 40 mL chloroform. After stirring and refluxing the mixture for 1 h, the unreacted CuCl was removed by filtration. The filtrate was then concentrated, and anhydrous ethanol was added, yielding $[CuPPh_3Cl]_4$ crystals.

Synthesis of $Cu_{13}(MBO)_{12}$ clusters

$Cu_{13}(MBO)_{12}$ (MBO = 2-mercaptobenzoxazole) was synthesized based on the reported literature [S5]. 30.0 mg $CuCl_2$ and 31.8 mg MBO were added into 10 mL acetonitrile. After vigorous stirring for 10 min, 10 mL of $NaBH_4$ in acetonitrile (0.6 mmol) was added. After reacting at room temperature for 1 h, the solvent was removed. The residue was washed with diethyl ether, and then extracted with dichloromethane to obtain the $Cu_{13}(MBO)_{12}$ clusters.

Synthesis of $Cu_{13}H_{10}(DCBT)_3(TFPP)_7$ clusters

$Cu_{13}H_{10}(DCBT)_3(TFPP)_7$ (DCBT = 2,4-dichlorobenzenethiol, TFPP = tris(4-fluorophenyl)phosphine) was synthesized based on the reported literature [S6]. 100 mg $Cu(CH_3CN)_4BF_4$ and 100 mg TFPP were added into 5 mL of a mixed acetonitrile-dichloromethane solvent (4:1, v/v). After complete dissolution, 30 μL DCBT was added and the mixture was stirred for 20 min. Then, 5 mL of a methanolic $NaBH_4$ solution (20 mg mL^{-1}) was introduced. The reaction was stirred at room temperature for 3 h, then the precipitate was collected, washed with methanol and *n*-hexane, and orange clusters were obtained.

Synthesis of $Cu_{14}H_{10}(DMBT)_3(PPh_3)_8$ clusters

$Cu_{14}H_{10}(DMBT)_3(PPh_3)_8$ (DMBT = 2,4-dimethylbenzenethiol) was synthesized based on the reported literature [S7]. 100 mg $Cu(CH_3CN)_4BF_4$ and 100 mg PPh_3 were added

into 5 mL of a mixed acetonitrile-chloroform solvent (4:1, v/v). After stirring for 30 min, 30 μL DMBT was added, and 5 mL of a methanolic $NaBH_4$ solution (20 mg mL^{-1}) was introduced. After stirring at room temperature for 1 h, the precipitate was removed, and the clear solution was kept in a refrigerator, yielding bright yellow clusters.

Synthesis of $Cu_{15}(PPh_3)_6(PET)_{13}$ clusters

$Cu_{15}(PPh_3)_6(PET)_{13}$ (PET = 2-phenylethanethiol) was synthesized based on the reported literature [S8]. 160 mg $Cu(CH_3CN)_4BF_4$ and 130 mg PPh_3 were added into 8 mL of a mixed acetonitrile-chloroform solvent (1:1, v/v). After complete dissolution, 50 μL PET was added and the mixture was stirred for 10 min. Then, 3 mL of a methanolic $NaBH_4$ solution (10 mg mL^{-1}) was introduced. After stirring at room temperature for 5 h, the solvent was removed. The residue was extracted with chloroform to obtain the $Cu_{15}(PPh_3)_6(PET)_{13}$ clusters.

Synthesis of $Cu_{16}(tBuA)_{12}(TFA)_4(MeOH)_2$ clusters

$Cu_{16}(tBuA)_{12}(TFA)_4(MeOH)_2$ (tBuA = tert-butylacetylene, TFA = CF_3COO) was synthesized based on the reported literature [S9]. 25.4 mg $Cu(CF_3COO)_2$, 12.7 mg Cu powder and 50 μL tBuA were added into 4 mL of methanol. After stirring at room temperature for 12 h, the precipitate was removed, and the clear solution was stored at 18 °C, yielding red needle-like cluster crystals.

Synthesis of $Cu_{25}H_{10}(DCBT)_{18}$ clusters

$Cu_{25}H_{10}(DCBT)_{18}$ was synthesized based on the reported literature [S10]. 100 mg $Cu(OAc)_2{\cdot}H_2O$ and 32 mg tetraphenylphosphonium tetraphenylborate were added into 6 mL of a mixed methanol-dichloromethane solvent (1:2, v/v). Then, 30 μL DCBT was added under vigorous stirring. After 20 min, 3 mL of $NaBH_4$ aqueous solution (30 mg mL^{-1}) was added. After reacting for 3 h, the solvent was removed, the residue was washed with a mixed methanol-H_2O solvent (4:1, v/v), and then extracted with dichloromethane to afford $Cu_{25}H_{10}(DCBT)_{18}$.

Synthesis of $Cu_{25}H_{22}(TFPP)_{12}$ clusters

$Cu_{25}H_{22}(TFPP)_{12}$ was synthesized based on the reported literature [S11]. 180 mg copper acetylacetonate was added into 50 mL of a mixed methanol-dichloromethane solvent (1:3, v/v). After vigorous stirring at 36 °C for 30 min, 300 mg of TFPP was quickly added. When the solution color changed from dark blue to light blue, 2 mL of an ice-cold $NaBH_4$ aqueous solution (75 mg mL^{-1}) was rapidly added. After reacting for 5 h, the solvent was removed, and the residue was washed with diethyl ether to afford $Cu_{25}H_{22}(TFPP)_{12}$.

Synthesis of $Cu_{28}(CHT)_{18}(PPh_3)_3$ clusters

$Cu_{28}(CHT)_{18}(PPh_3)_3$ (CHT = cyclohexanethiol) was synthesized based on the reported literature [S12]. 100 mg $Cu(CH_3CN)_4BF_4$ and 80 mg PPh_3 were added into 5 mL of a mixed acetonitrile-dichloromethane solvent (4:1, v/v). After 20 min, 5 mL of a methanolic $NaBH_4$ solution (20 mg mL^{-1}) was introduced. After reacting for 3 h, the precipitate was collected by centrifugation and washed with methanol and *n*-hexane to afford $Cu_{28}(CHT)_{18}(PPh_3)_3$.

Synthesis of $Cu_{36}H_{10}(PET)_{24}(PPh_3)_6$ clusters

$Cu_{36}H_{10}(PET)_{24}(PPh_3)_6$ was synthesized based on the reported literature [S13]. 100 mg $Cu(CH_3CN)_4BF_4$, 100 mg PPh_3 and 30 μL PET were added into 5 mL of a mixed acetonitrile-chloroform solvent (4:1, v/v). After 5 min, 5 mL of a methanolic $NaBH_4$ solution (20 mg mL^{-1}) was introduced. After reacting for 1 h, the precipitate was collected by centrifugation and washed with methanol and *n*-hexane to afford $Cu_{36}H_{10}(PET)_{24}(PPh_3)_6$.

Synthesis of $Cu_{53}(tBuA)_{20}(TFA)_{10}Cl_2H_{18}$ clusters

$Cu_{53}(tBuA)_{20}(TFA)_{10}Cl_2H_{18}$ was synthesized based on the reported literature [S14]. 12.7 mg $Cu(CF_3COO)_2$, 6.35 mg Cu powder and 42 μL tBuA were added into 16 mL of a mixed dichloromethane-methanol solvent (3:1, v/v). After stirring at room temperature for 3 h, 8.2 mg Ph_2SiH_2 was added. After reacting for 4 h, the supernatant

was collected by centrifugation and allowed to stand for 4 days to obtain red cluster crystals.

Catalytic tests

Preparation of the working electrode: 1.0 mg cluster and 9.0 mg multi-walled carbon nanotubes were added into a small amount of *N,N*-Dimethylformamide (DMF). After the turbid liquid was ultrasonicated for 30 min, the solvent was removed to obtain supported catalyst $Cu_4Pt_2(C\equiv CCyOH)_8$/CNT (short for Cu_4Pt_2). Then, 10 mg catalyst was dispersed in 5 mL isopropanol/H_2O (3:1, v/v) mixture by sonication to form a uniform catalyst ink (containing 50 μL 0.5 wt% Nafion solution). Finally, 500 μL catalyst ink was dropped on the surface of carbon paper (1 cm × 1 cm), and dried naturally to serve as the working electrode.

All electrochemical tests were performed in a H-type electrolytic cell at the electrochemical workstation CHI660E (Shanghai Chenhua) using a standard three-electrode system with catalyst-coated carbon paper as the working electrode, platinum wire as the counter electrode, and Ag/AgCl as the reference electrode. The anodic electrolyte was 0.3 M tetrabutylammonium hexafluorophosphate ($TBAPF_6$) in acetone containing 100 mM toluene, while the cathodic electrolyte was 0.1 M H_2SO_4 aqueous solution. The anode and cathode compartments were separated by a Nafion 117 membrane. The linear scanning voltammetry (LSV) curve was tested in a potential range of 0 to 2.3 V *vs.* Ag/AgCl, and the sweep rate was 10 mV s^{-1}. Chronoamperometry test was performed at different potentials for 1 h and the electrolytes were collected to calculate the Faraday efficiency and yield rate of benzaldehyde (BA).

In situ electrochemical impedance spectroscopy (EIS): the potential range was 1.5~2.1 V (*vs.* Ag/AgCl), the frequency range was 10^5 to 10^{-2} Hz and an amplitude was 5 mV. The electrolyte was 0.3 M $TBAPF_6$ in acetone containing 100 mM toluene.

Fourier-transformed alternating current voltammetry (FTACV): the potential range was 0.3~2.3 V (*vs.* Ag/AgCl), amplitude was 0.1 V, and the alternating frequency was 10 Hz.

Catalytic products were analyzed by gas chromatography-mass spectrometry (SHIMADZU, GC-2030AM, 230 V) and ^{1}H nuclear magnetic resonance spectroscopy (Bruker Advance II 400 MHz). The Faraday efficiencies and yield rates of the products were calculated according to the formula:

$$FE_{BY} = \frac{2F \times n_{BY}}{Q} \times 100\%$$

$$FE_{BA} = \frac{4F \times n_{BA}}{Q} \times 100\%$$

$$FE_{BO} = \frac{6F \times n_{BO}}{Q} \times 100\%$$

Where F represented the Faradic constant 96485 C mol^{-1}, Q represented the total consuming amount of charge, n_{BY}, n_{BA} and n_{BO} represented the formed molar amount of benzyl alcohol, benzaldehyde and benzoic acid, respectively.

Characterization

Electrochemical surface area (ECSA) test: Cyclic voltammetry (CV) was performed within a non-faradaic potential window of 0.3-0.4 V (*vs.* Ag/AgCl) at scan rates of 20, 40, 60, 80, 100, and 120 mV s^{-1}. The double-layer capacitance (C_{dl}) was determined by linear fitting of double-layer capacitance. The ECSA was then calculated using the formula: ECSA = C_{dl}/C_s, where Cs represents the specific capacitance of a flat surface under identical conditions, taken here as 40 μF cm^{-2}.

The UV-vis spectra were recorded on a Shimadzu UV-1800 UV-vis spectrometer.

The single crystal X-ray diffraction data of $Cu_4Pt_2(C{\equiv}CCyOH)_8$ were collected on a Bruker D8 VENTURE with Mo Kα radiation (λ = 0.71073 Å) at 193 K.

Electrospray ionization mass spectrometry (ESI-MS) measurements were performed on a Thermo Fisher Scientific Q Exactive combined Quadrupole-Orbitrap Mass Spectrometer. $Cu_4Pt_2(C{\equiv}CCyOH)_8$ was dissolved in the DMF/MeOH (1:9, v/v) mixture containing 5 μL of acetic acid, with the cluster concentration of 0.5 mg mL^{-1}.

Scanning tunneling microscopy break junctions (STM-BJ) technique was used to measure the current through a cluster trapped between an Au tip and the gold substrate. The Au tip was made from gold wire (99.999%, 0.25 mm diameter). The substrates were Au (111) films on glass wafers. Prior to each measurement session, a blank calibration was conducted using a pure solvent mixture of tetrahydrofuran (THF) and 1,3,5-trimethyl (TMB) (1:1, v/v) without any clusters. This procedure ensured a clean junction environment and allowed for the calibration of the stretching speed and the Au-Au snap-back distance. One end of a gold wire (diameter 0.25 mm, purity 99.999%) was melted in a flame to form a microscale gold sphere, which served as the STM probe tip. The cluster was dissolved in the THF:TMB mixture to prepare a 0.1 mM solution. A constant bias potential (V bias) of 100 mV was applied between the gold substrate and the gold tip. The experiments were carried out at room temperature in air. A gold tip moved up and down to touch the gold substrate, leading to the continual formation and breaking of the junction. When the gold tip moved up, a single gold atom junction (the conductance quantum G_0) was formed due to the ductile nature of the gold. When it continued to open, the Au-Au contact fractured and formed a nanogap between the tip and the substrate; then the cluster entered the gap to form a single cluster junction. Thousands of single-cluster junctions were constructed by moving the tip up and down repeatedly, while the current versus length traces were recorded. The distance between the tip and substrate was the sum of the length of conductance junction, and an Au-Au snap back distance (0.5 nm). The conductance measurements for the clusters were carried out using STM-BJ with 0.1 V bias dissolved in the THF:TMB mixture.

X-ray photoelectron spectra (XPS) were collected on the RBD upgraded PHI 5000C ESCA System in a high-vacuum chamber with the base pressure below 1×10^{-8} Torr. All binding energies were calibrated using the C1s peak (284.8 eV).

Powder X-ray diffraction (XRD) patterns of catalysts were collected on a Bruker D8 Advance diffractometer with a Cu Kα source (λ = 0.1541 nm) at a scanning speed of $0.1°\ s^{-1}$ from 5° ~ 60° and operated at 40 kV, 40 mA.

Transmission electron microscope (TEM) and energy dispersive spectroscopy mapping (EDS mapping) images of samples were taken on a JEM-2100F operated at a voltage of 200 kV.

Electron paramagnetic resonance (EPR) spectroscopy was performed on a Bruker A300 spectrometer at room temperature. The in situ electrochemical EPR tests employed a three-electrode H-cell separated by a Nafion 117 membrane. The working electrode was prepared by coating the catalyst onto a 1 cm × 1 cm carbon paper, while an Ag/AgCl electrode and a platinum sheet were used as the reference and counter electrodes, respectively. Prior to the test, 200 μL of 5,5-dimethyl-1-pyrroline-N-oxide (DMPO) was added to 15 mL of the electrolyte as a spin trap. After electrolysis at 2.0 V (*vs.* Ag/AgCl), 30 μL of the solution was transferred to a quartz sample tube, the bottom of which was sealed with a wax plug. The tube was then inserted into the EPR-specific sample tube for radical detection.

Radical quenching experiments were performed in the presence of various radical scavengers (10 mM) (tert-butanol (TBA) as •OH radical scavenger, butylated hydroxytoluene (BHT) as carbon-centered radical scavenger, benzoquinone (BQ) as $•O_2^-$ scavenger) in acetone electrolyte containing 0.3 M $TBAPF_6$ with 100 mM toluene at 1.8 V *vs.* Ag/AgCl for 20 min.

Time-resolved in situ transmission Fourier Transform Infrared (FT-IR) spectroscopy was conducted on a Thermo Scientific Nicolet iS50 FTIR instrument. Firstly, the catalyst was homogeneously mixed with KBr, thoroughly ground, and then pressed into a disk. Before measurement, the sample was pretreated in N_2 for 30 min at room temperature to remove impurities on the sample surface. After the background spectrum was recorded, 50 μL toluene was applied to the sample surface, allowed to equilibrate for 5 minutes, and then purged with N_2 at a flow rate of 30 mL min^{-1}. The IR spectra were continuously collected until the characteristic infrared bands of toluene had completely disappeared. Each spectrum was acquired by averaging 64 scans at a resolution of 4 cm^{-1}.

In situ attenuated total reflection Fourier transform infrared (ATR-FTIR) spectroscopy was performed on a Thermo Scientific Nicolet iS50 spectrometer equipped with a liquid nitrogen-cooled MCT detector. The working electrode was prepared by dropping the catalyst ink onto a silicon ATR crystal. A standard three-electrode configuration was employed, utilizing a Ag/AgCl reference electrode and a platinum sheet counter electrode. The electrochemical cell, coupled to the FTIR system, contained 0.3 M $TBAPF_6$ in acetone with 100 mM toluene as the anodic electrolyte, separated by a Nafion 117 membrane from the cathodic compartment filled with 0.1 M H_2SO_4 aqueous solution. Spectra were acquired under applied potentials ranging from 1.0 V to 2.2 V (*vs.* Ag/AgCl) using a CHI660E electrochemical workstation (Shanghai Chenhua).

In-situ Raman spectroscopy measurements were performed with a 633 nm laser and the 50x objective lens on the Horiba LabRAM Evolution Raman microscope equipped with a flow cell. The acquisition time of each test was 5 s, and the average signal of three scans was recorded. The catalyst ink was uniformly dropped onto the glassy carbon electrode of Raman electrochemical cell as the working electrode. Ag/AgCl electrode was used as the reference electrode, and platinum wire was used as the counter electrode. The anodic electrolyte (0.3 M $TBAPF_6$ in acetone with 100 mM toluene) was circulated through the anode chamber at 5 mL min^{-1} via a peristaltic pump. The spectra were collected under applied potentials ranging from 1.0 V to 2.2 V (*vs.* Ag/AgCl) using a CHI760E electrochemical workstation (Shanghai Chenhua).

2. Computational Details

All density functional theory (DFT) calculations were carried out using the Vienna ab initio simulation package (VASP 5.4.4) [S15,S16]. The generalized gradient approximation (GGA) with Perdew-Burke-Ernzerhof (PBE) functional was adopted to describe the exchange correlation energy [S17], while the ion-core electronic interactions were treated by the projected augmented wave (PAW) method [S18]. The Pt(111) surface was modelled by $p(4 \times 4)$ supercell with four atomic layers, the bottom two layers are fixed, whereas the rest atoms are allowed to relax. A vacuum region of 15 Å was set between each slab to avoid any periodic interactions. A plane wave basis

with a kinetic energy cutoff of 400 eV was used and the DFT-D3 method was employed to calculated the van der Waals interactions [S19]. The Brillouin zone was sampled with a gamma k-point for geometry optimization. The convergence criteria for atomic relaxation were 1×10^{-5} eV of energy and 0.03 eV $Å^{-1}$ of force, respectively. The adsorption energy (E_{ads}) of adsorbents on the cluster was determined by $E_{ads} = E_{x/cluster} - E_{cluster} - E_x$, where $E_{x/cluster}$ refers to the total energy of the $Cu_4Pt_2(C{\equiv}CCyOH)_8$ cluster with adsorbed molecules (x), $E_{cluster}$ and E_x represent the total energy of clean $Cu_4Pt_2(C{\equiv}CCyOH)_8$ cluster and free molecule, respectively.

3. Supporting Figures and Tables

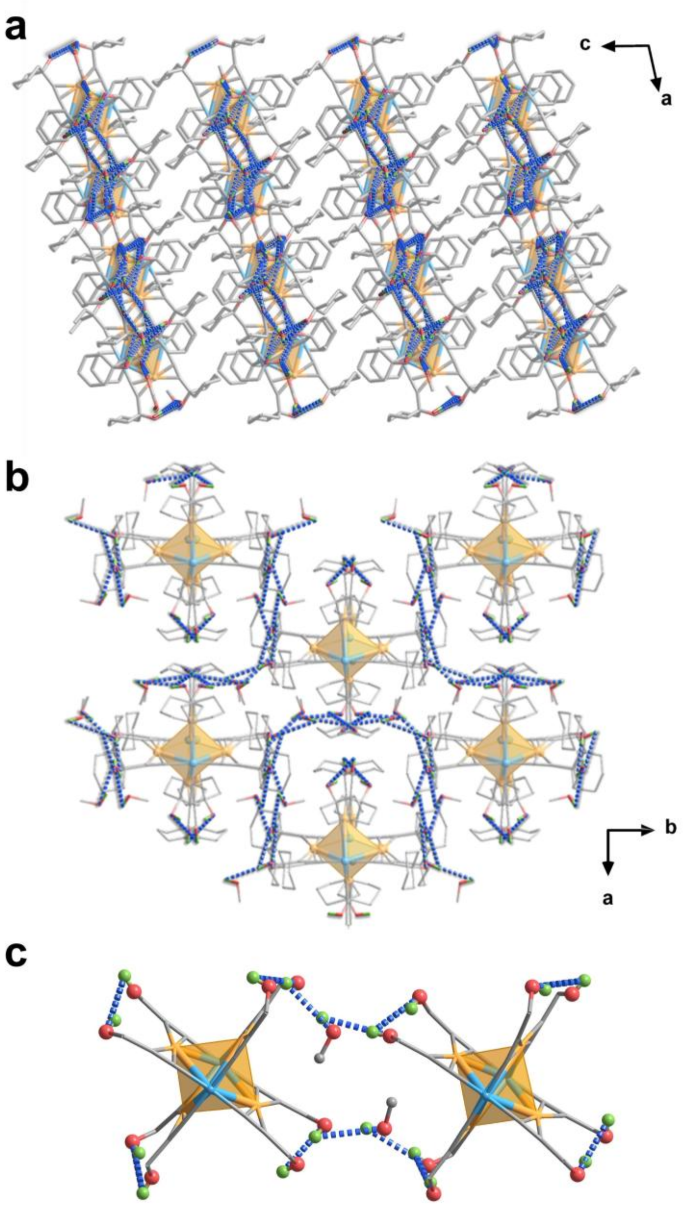

Figure S1. 2D hydrogen-bonding network formed via methanol bridges. (a) Packing pattern viewed along *b*-axis. (b) Packing pattern viewed along *c*-axis. (c) Schematic illustration of hydrogen bonding within a single cluster and between adjacent clusters. Color code: orange, Cu; light blue, Pt; red, O; grey, C; green, H; dark blue, H-bond. All H atoms bonded to carbon are omitted for clarity.

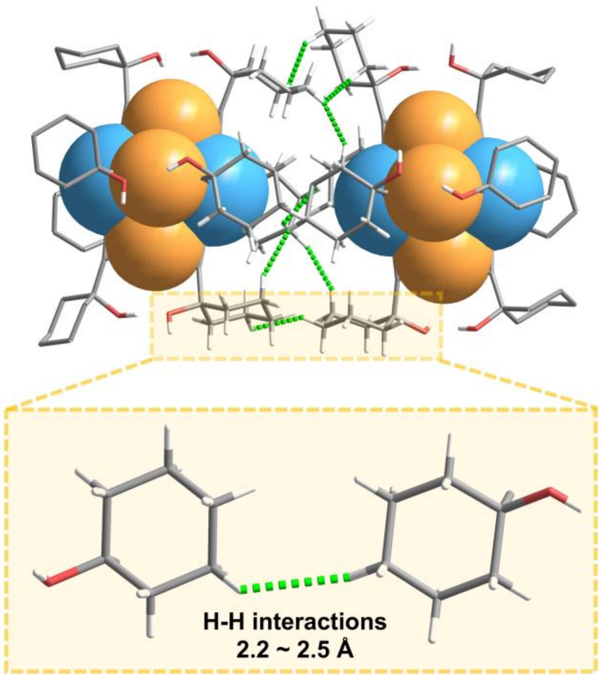

Figure S2. Weak interlayer supramolecular interactions (H···H interactions).

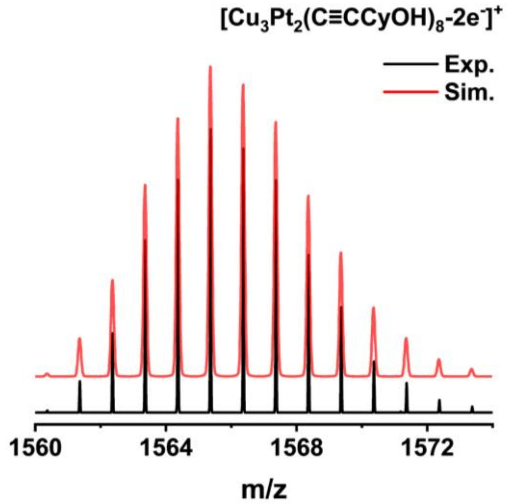

Figure S3. Comparison of the experimental (black) and simulated (red) isotopic patterns for $[Cu_3Pt_2(C{\equiv}CCyOH)_8\text{-}2e^-]^+$.

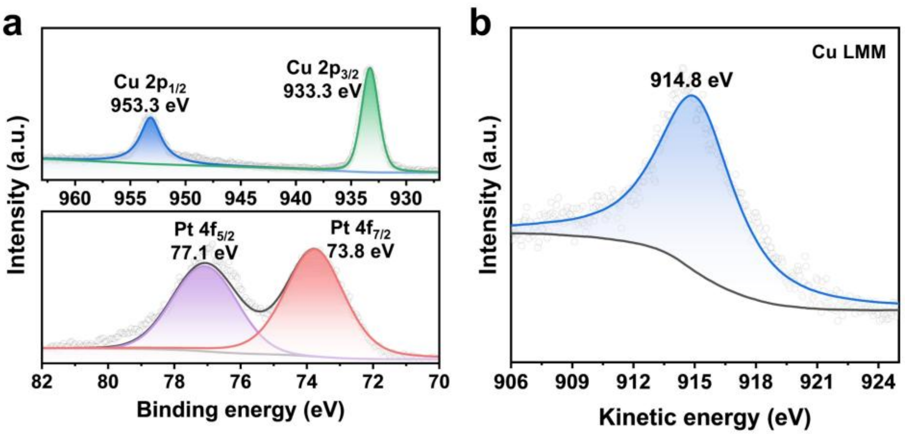

Figure S4. (a) Cu 2p and Pt 4f XPS profiles of $Cu_4Pt_2(C{\equiv}CCyOH)_8$. (b) Cu LMM spectra of $Cu_4Pt_2(C{\equiv}CCyOH)_8$.

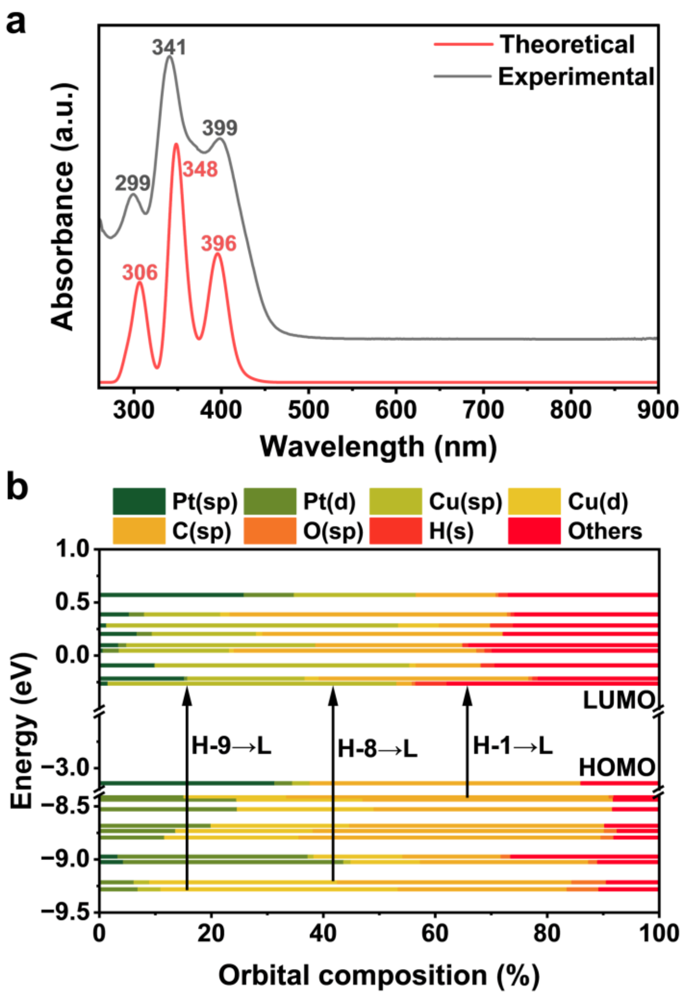

Figure S5. (a) Experimental and theoretical absorption spectra for $Cu_4Pt_2(C{\equiv}CCyOH)_8$. (b) KS orbital energy level diagram for $Cu_4Pt_2(C{\equiv}CCyOH)_8$ with contributions from various atomic-orbitals of Cu, Pt, C, O and H.

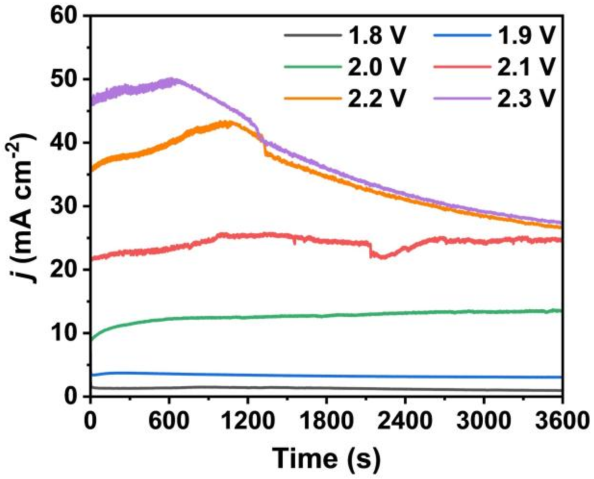

Figure S6. Current-time curves of Cu_4Pt_2 catalyst at different potentials. The electrolyte solution was 0.3 M $TBAPF_6$ in acetone containing 100 mM toluene.

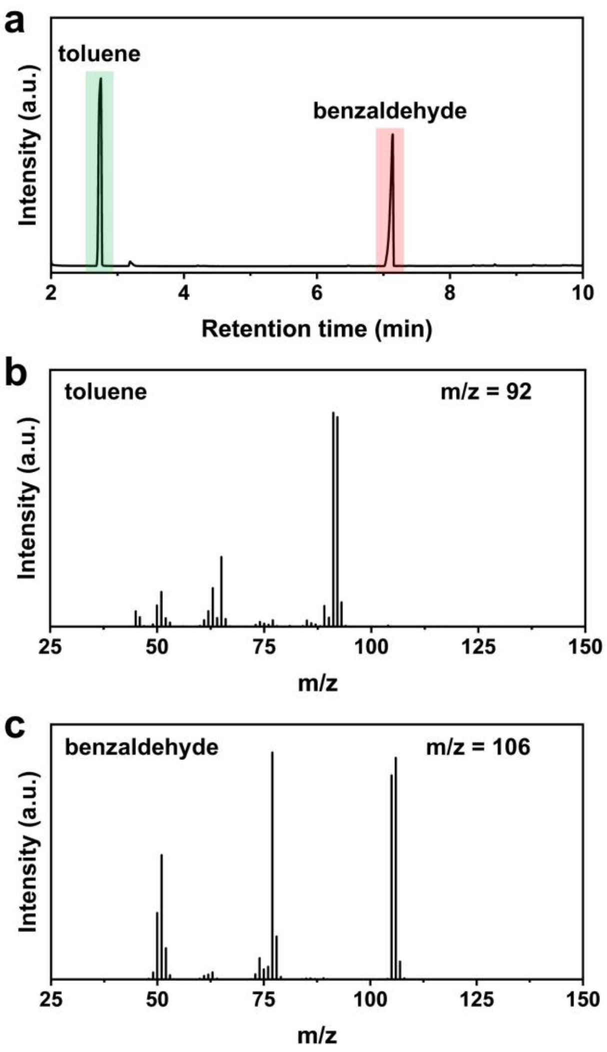

Figure S7. GC-MS results of the post-reaction anolyte over the Cu_4Pt_2 catalyst. (a) GC-FID data of the products from toluene oxidation over Cu_4Pt_2 catalyst. (b) MS data from toluene substrate. (c) MS data of benzaldehyde product.

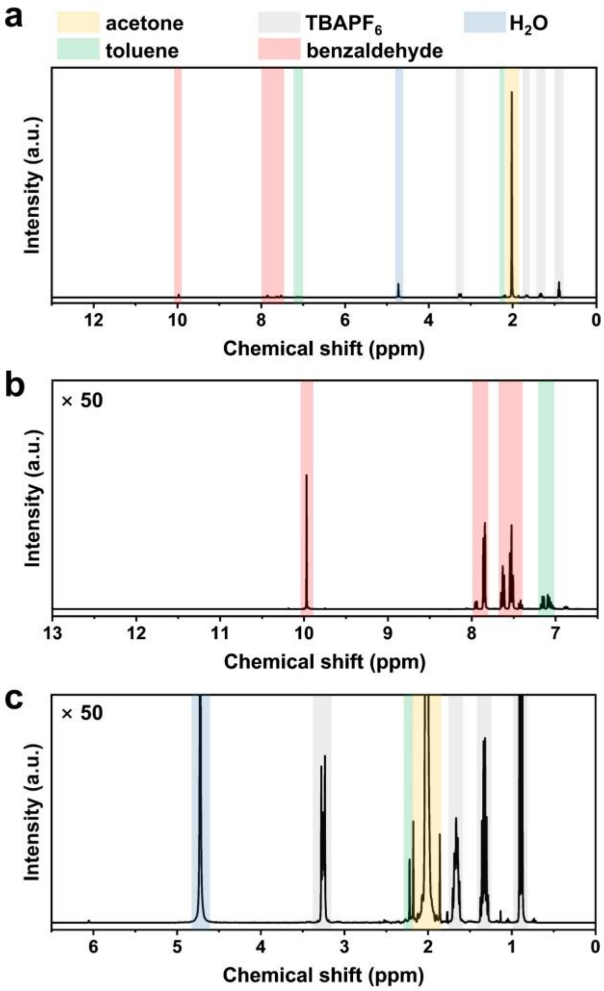

Figure S8. ^{1}H NMR spectra of the post-reaction anolyte after 9 h of chronoamperometric testing over the Cu_4Pt_2 catalyst. (a) Full spectrum. (b), (c) Magnified views.

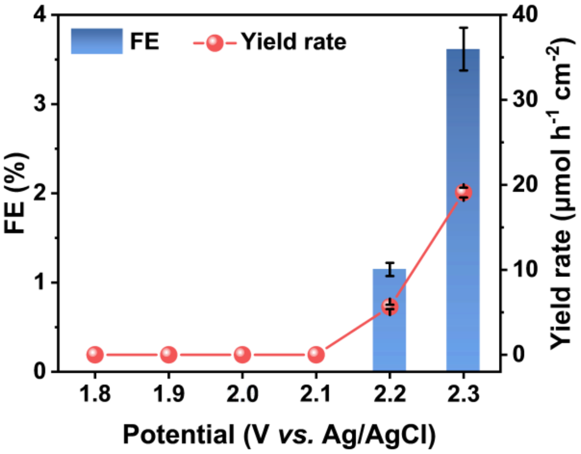

Figure S9. Potential-dependent yield rates and FEs of benzoic acid on the Cu_4Pt_2 catalyst.

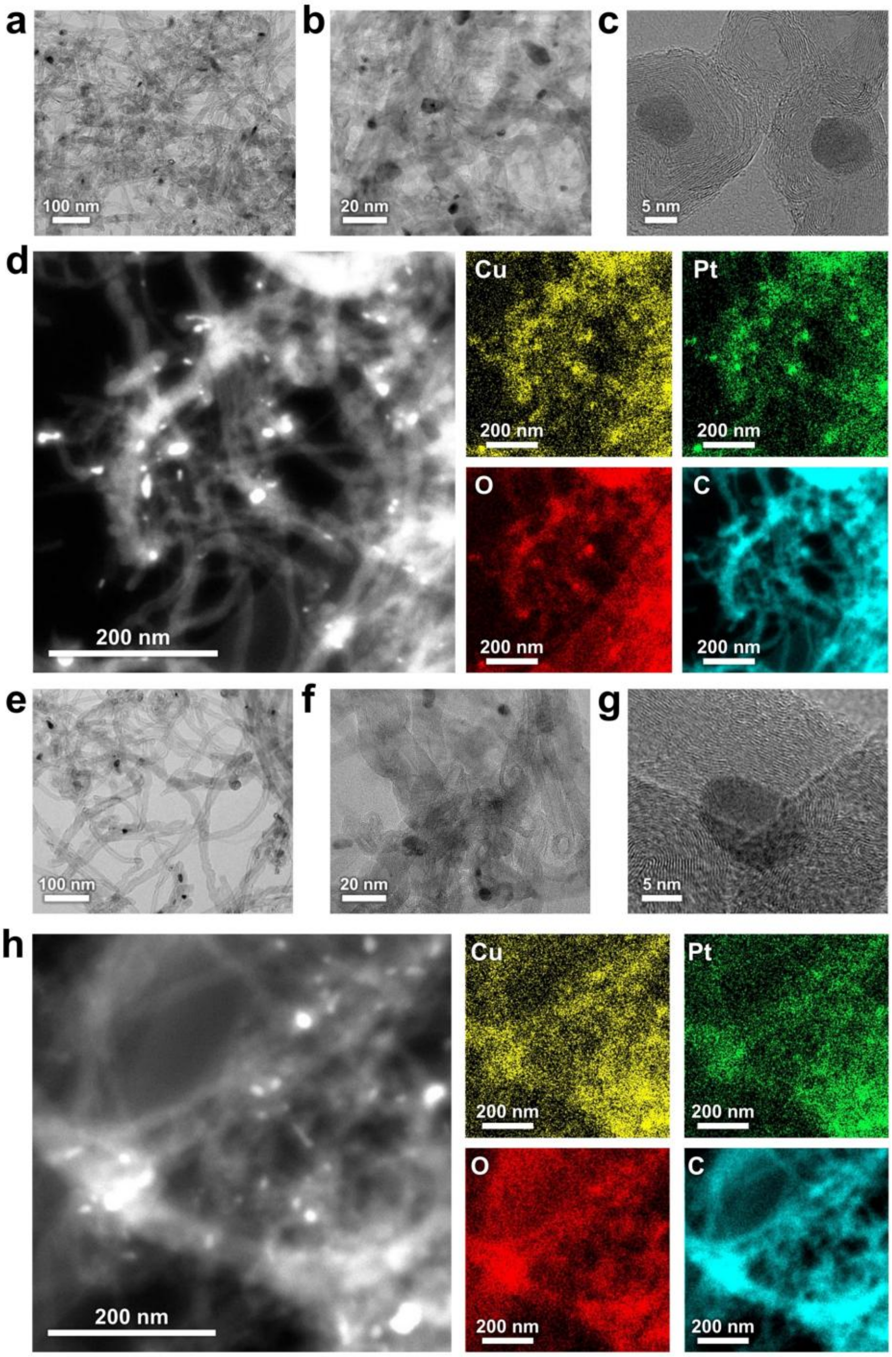

Figure S10. (a), (b), (c) TEM images and high-resolution TEM (HR-TEM) images of Cu_4Pt_2 clusters loaded on the carbon nanotubes before reaction. (d) High-angle annual dark-filed scanning transmission electron microscopy images and STEM-EDS elemental mappings of Cu_4Pt_2 clusters before reaction. (e), (f), (g) TEM images and HR-TEM images of Cu_4Pt_2 clusters loaded on the carbon nanotubes after reaction. (h) High-angle annual dark-filed scanning transmission electron microscopy images and STEM-EDS elemental mappings of Cu_4Pt_2 cluster after reaction.

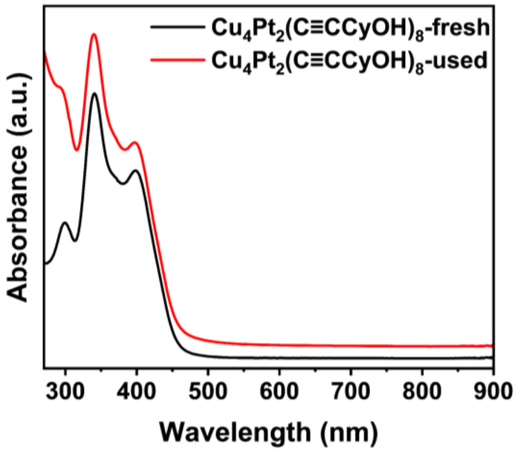

Figure S11. UV-vis absorption spectra of the $Cu_4Pt_2(C{\equiv}CCyOH)_8$ clusters before reaction and after reaction.

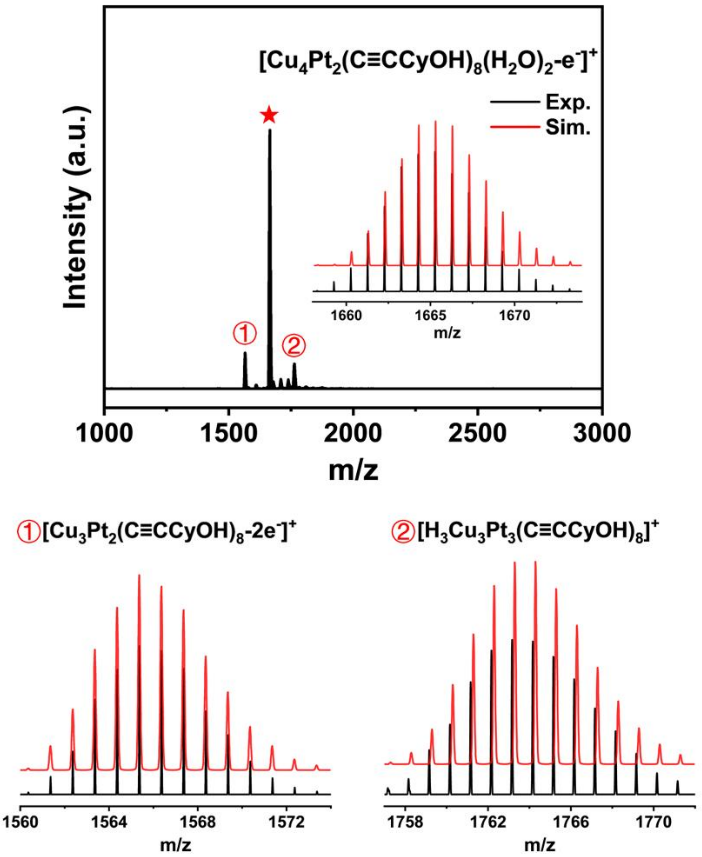

Figure S12. ESI-MS spectra of $Cu_4Pt_2(C{\equiv}CCyOH)_8$ after reaction. Comparison of the experiment (black) and simulated (red) isotopic patterns for $[Cu_4Pt_2(C{\equiv}CCyOH)_8(H_2O)_2\text{-}e^-]^+$, $[Cu_3Pt_2(C{\equiv}CCyOH)_8\text{-}2e^-]^+$ and $[H_3Cu_3Pt_3(C{\equiv}CCyOH)_8]^+$.

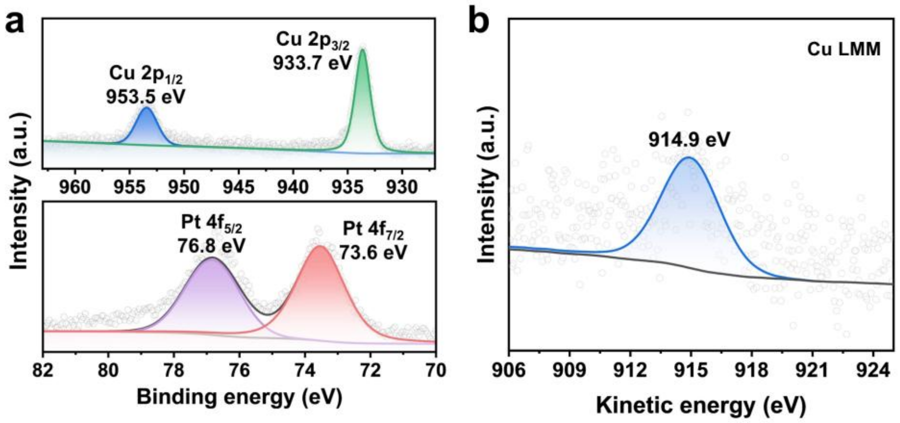

Figure S13. (a) Cu 2p and Pt 4f XPS profiles of Cu_4Pt_2 after reaction. (b) Cu LMM spectra of Cu_4Pt_2 after reaction.

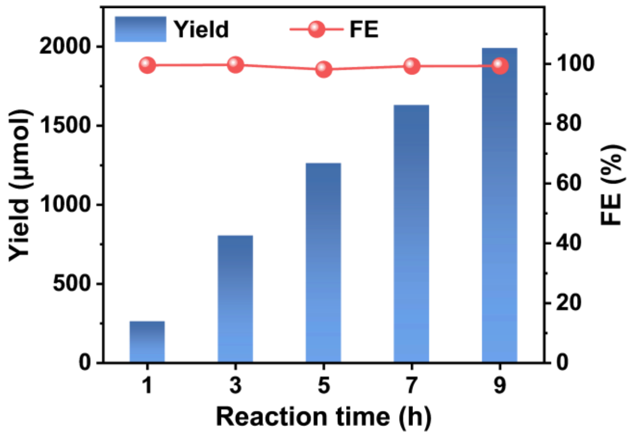

Figure S14. FEs and yield rates of benzaldehyde over Cu_4Pt_2 as a function of reaction time.

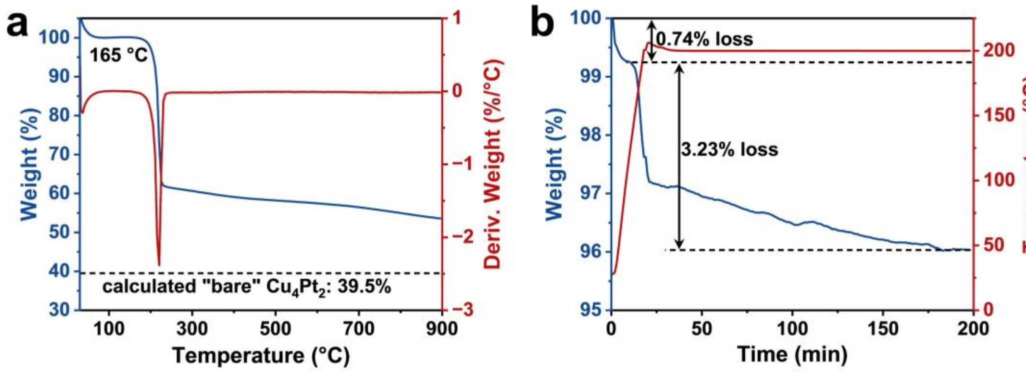

Figure S15. (a) Simultaneous TG-DSC analysis of the Cu_4Pt_2 catalyst. Thermogravimetric (TG, blue) and differential scanning calorimetry (DSC, red) curves measured under N_2 atmosphere at a heating rate of 10 °C min^{-1}. The DSC peaks revealed the exothermic or endothermic nature of the decomposition steps observed in the TG curve. (b) TG curves of Cu_4Pt_2 clusters supported on carbon nanotubes recorded under N_2 atmosphere with the temperature first ramped at 10 °C min^{-1} to 200 °C, followed by an isothermal hold at 200 °C.

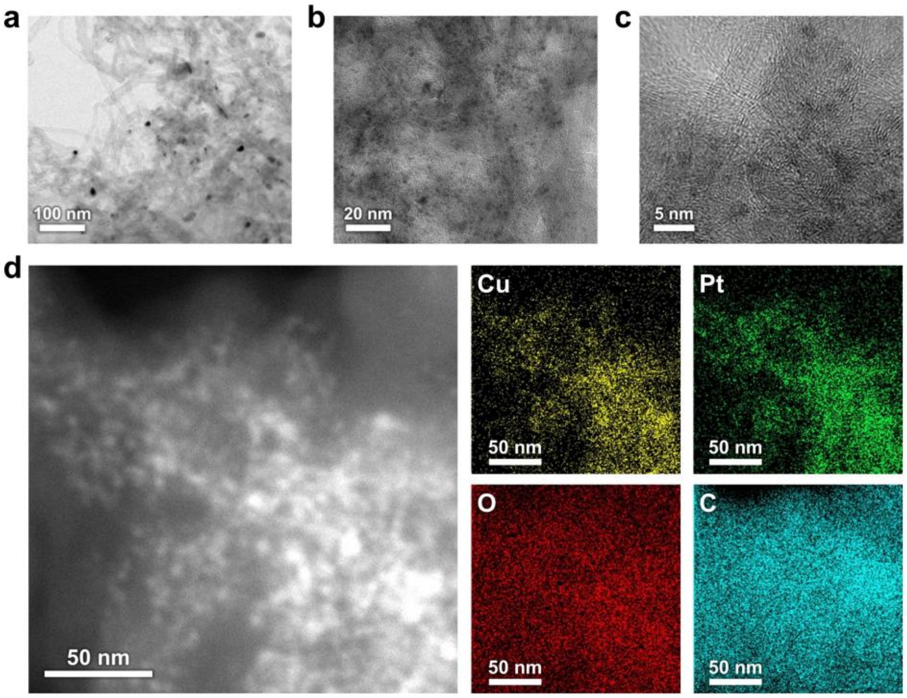

Figure S16. (a), (b), (c) TEM images and high-resolution TEM (HR-TEM) images of Cu_4Pt_2-Calc sample. (d) High-angle annual dark-filed scanning transmission electron microscopy images and STEM-EDS elemental mappings of Cu_4Pt_2-Calc sample.

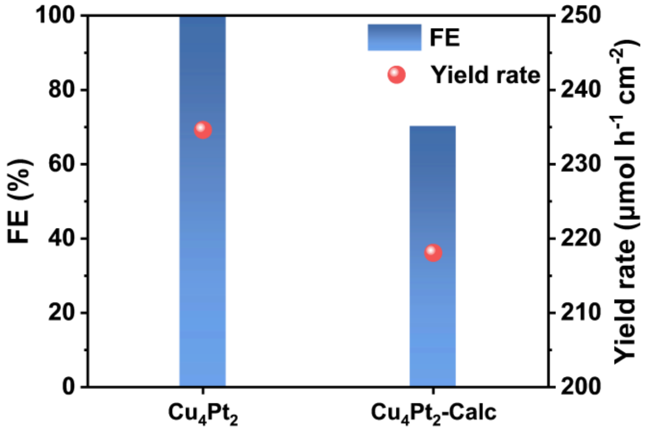

Figure S17. Comparison of FEs and yield rates of benzaldehyde at 2.1 V *vs.* Ag/AgCl between Cu_4Pt_2 cluster and Cu_4Pt_2-Calc sample.

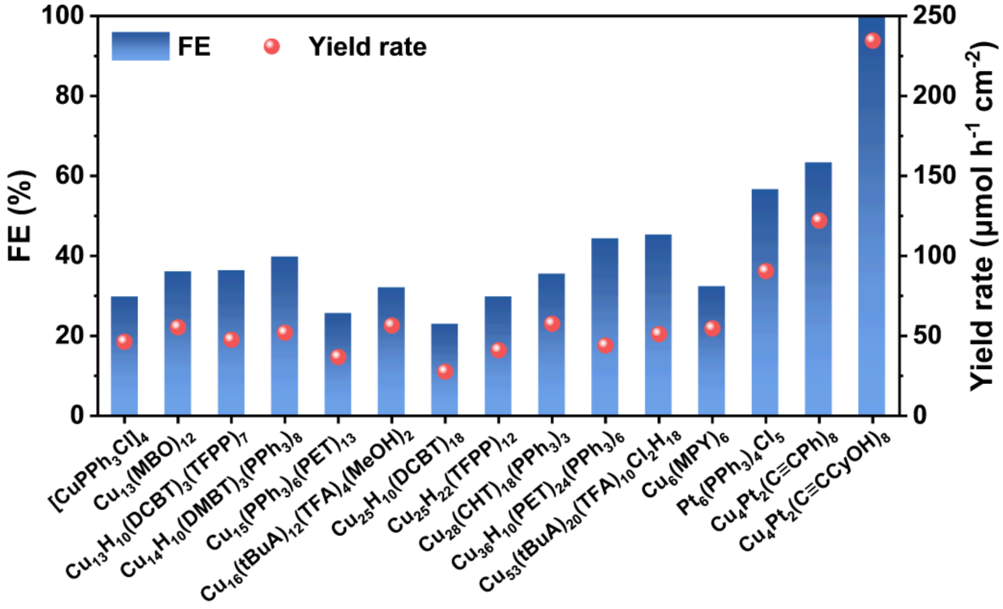

Figure S18. Comparison of FEs and yield rates of benzaldehyde obtained from a series of Cu-based cluster catalysts at a potential of 2.1 V *vs.* Ag/AgCl under other identical conditions.

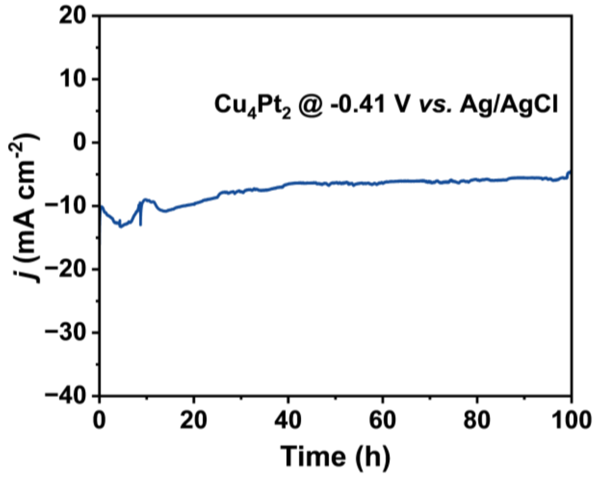

Figure S19. Long-term stability at -0.41 V *vs.* Ag/AgCl in 0.1 M H_2SO_4 electrolyte.

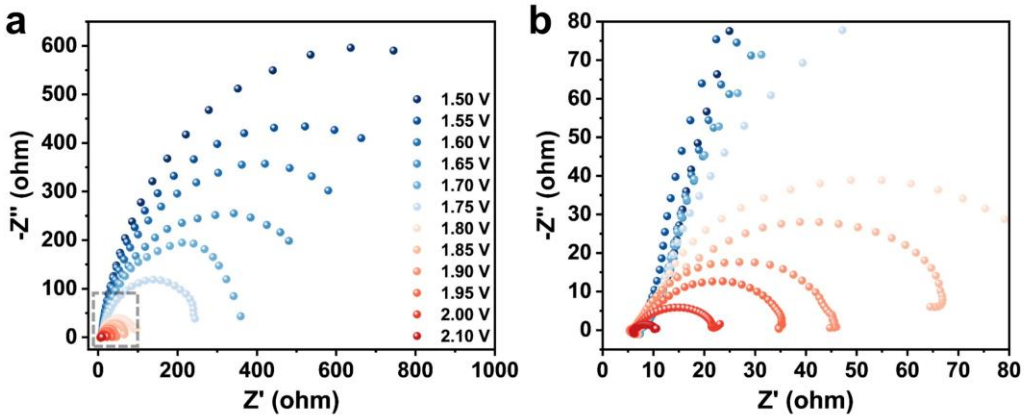

Figure S20. (a) The potential-dependent Nyquist plots over Cu_4Pt_2. (b) Enlarged section of the high-frequency region shown in the grey box of a.

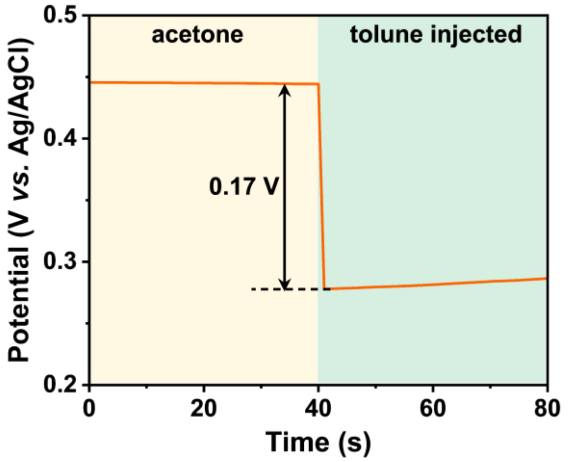

Figure S21. The OCP of Cu_4Pt_2 in acetone electrolyte (0.3 M $TBAPF_6$) before and after toluene was injected.

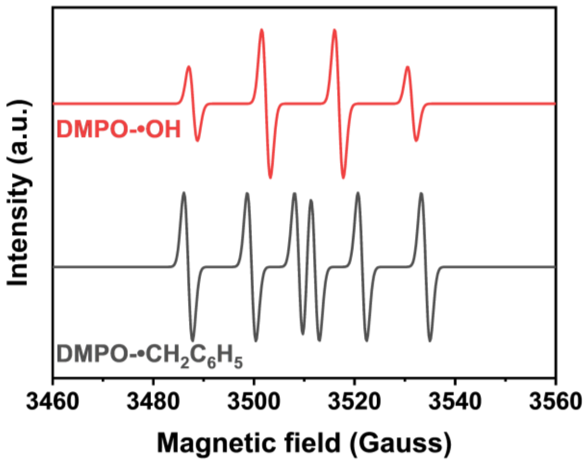

Figure S22. Simulated EPR spectra obtained from DMPO-trapped •OH and •$CH_2C_6H_5$.

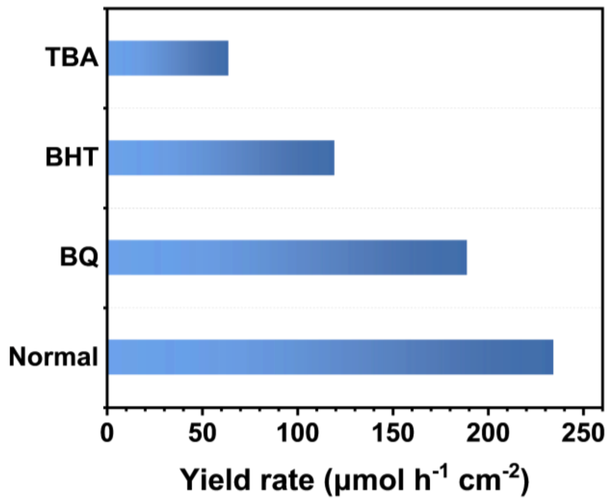

Figure S23. Quenching experiments of toluene oxidation over Cu_4Pt_2.

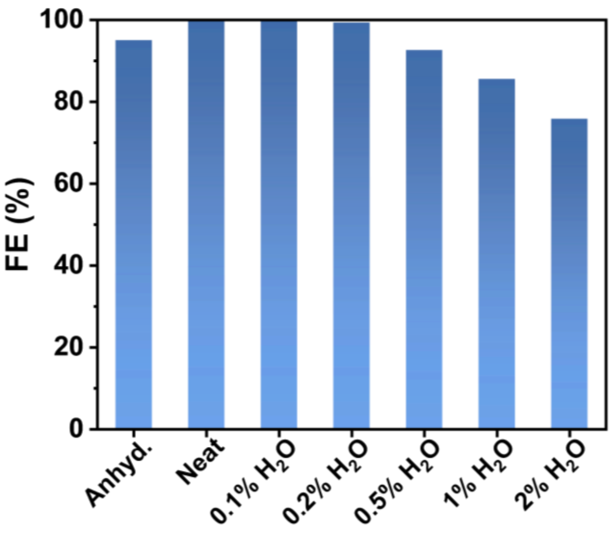

Figure S24. Effects of water content in the acetone electrolyte on the Faradaic efficiency for toluene oxidation to benzaldehyde. Anhyd.: anhydrous acetone; Neat: acetone stored under conventional conditions; 0.1% H_2O, 0.2% H_2O, 0.5% H_2O, 1% H_2O, and 2% H_2O: acetone with the additional water based on the volume content.

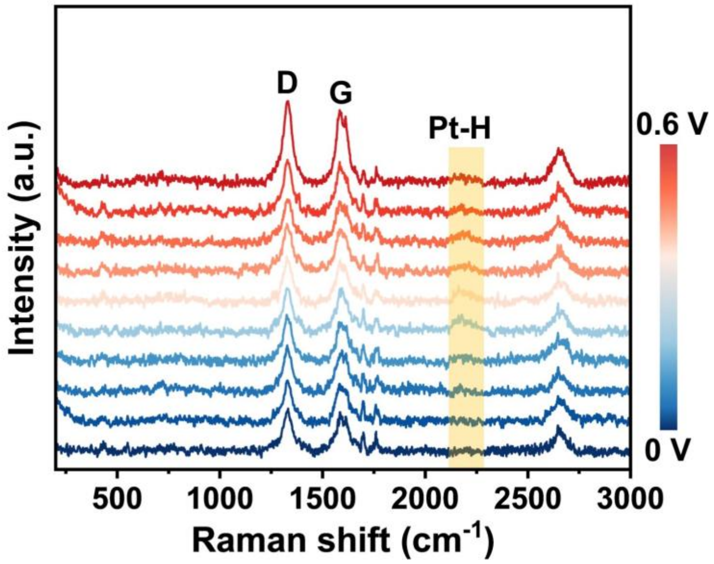

Figure S25. In situ Raman spectra of Cu_4Pt_2 recorded in 0.1 M H_2SO_4 electrolyte, at applied potentials from 0 to 0.6 V *vs*. Ag/AgCl.

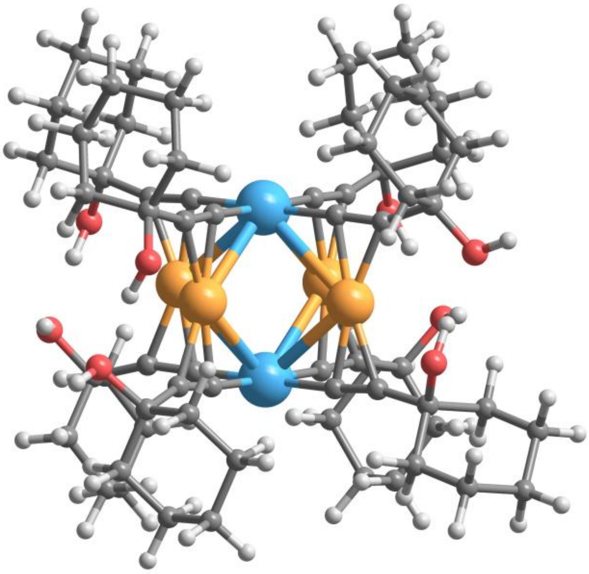

Figure S26. DFT-optimized structure of $Cu_4Pt_2(C{\equiv}CCyOH)_8$ cluster. Color code: orange, Cu; light blue, Pt; red, O; grey, C; white, H.

Table S1. Crystal data and structure refinement for the $Cu_4Pt_2(C{\equiv}CCyOH)_8$ cluster.

Empirical formula	C67H100Cu4O11Pt2	
Formula weight	1725.80	
Temperature	193.0 K	
Wavelength	0.71073 Å	
Crystal system	monoclinic	
Space group	P-1	
Unit cell dimensions	a = 13.9992(5) Å	α = 90°
	b = 21.3655(8) Å	β = 101.678(1)°
	c = 23.4954(8) Å	γ = 90°
Volume	6882.0(4) $Å^3$	
Z	4	
Density (calculated)	1.666 Mg/m^3	
Absorption coefficient	5.319 mm^{-1}	
F(000)	3448	
Theta range for data collection	2.602 to 27.602°	
Index ranges	-18<=h<=14, -27<=k<=27, -29<=l<=30	
Reflections collected	62289	
Independent reflections	15876 [R(int) = 0.0643, R(sigma)= 0.0562]	
Completeness to theta = 25.242°	99.2 %	
Absorption correction	Multi-Scan	
Refinement program	SHELXL-2018/3(Sheldrick,2018)	
Data / restraints / parameters	15876 / 1398 / 772	
Goodness-of-fit on F^2	1.056	
Δ/σ_{max}	0.001	
Final R indices [I>2sigma(I)]	R1 = 0.0461, wR2 = 0.0847	
R indices (all data)	R1 = 0.0684, wR2 = 0.0952	
Largest diff. peak and hole	1.232 and -1.389 $eÅ^{-3}$	

Table S2. Performance comparison of Cu_4Pt_2 with reported electrocatalysts for toluene oxidation to benzaldehyde.

Anolyte	Working electrode	Condition	yield rate (μmol h^{-1} cm^{-2})	FE (%)	Selectivity (%)	References
0.3 M $TBAPF_6$	Cu_4Pt_2	ambient	234.6	99.62	100	This work
0.06 M $LiNO_3$, 0.1 M $LiBF_4$ in 4:1 MeCN:HFIP	CoO_x/IrO_2	Air	200.5	86	100	[S20]
0.1 M $LiBF_4$ in 9:1 MeCN:HFIP	Graphite rod	ambient	55.97	60	92.4	[S21]
5 g/L NaOH: acetic acid = 1/5	Co_3O_4@MnO_2	Air 80 °C	12.1	65	92	[S22]
0.05 M $TBANO_3$ in MeCN	Glassy carbon	1 atm O_2	7.46	8	37	[S23]
0.3 M $TBAClO_4$ in CH_3CN	$HPMoV_2$/CP	ambient	-	-	83.5	[S24]
0.5 M H_2SO_4	CC/PANI/G	ambient	-	-	44.9	[S25]
50% H_2SO_4	$H_5PV_2Mo_{10}O_{40}$ • $32H_2O$	N_2 70 °C	-	-	95	[S26]
AcOH	Co(II)	1 atm O_2 85 °C	-	-	74	[S27]
0.5 M Na_2SO_4	$rGO/PtO_x/TiO_2$	ambient 100 mW cm^{-2} light	1.75	-	83.5	[S28]

4. Supporting References

S1. Dong J-P, Xu Y, Zhang X-G *et al*. Copper-sulfur-nitrogen cluster providing a local proton for efficient carbon dioxide photoreduction. *Angew Chem Int Ed* 2023; **62**: e202313648.

S2. Wang X, Zhao L, Li X *et al*. Atomic-precision Pt_6 nanoclusters for enhanced hydrogen electro-oxidation. *Nat Commun* 2022; **13**: 1596.

S3. Yam VWW, Yu KL, Cheung KK. Luminescence and aggregation studies of hexanuclear platinum-copper acetylide complexes. Crystal structure of the luminescent metal-metal bonded dimer $[Pt_2Cu_4(C{\equiv}CPh)_8]_2$. *J Chem Soc Dalton Trans* 1999; 2913-2915.

S4. Churchill MR, Bezman SA, Osborn JA *et al*. Synthesis and molecular geometry of hexameric triphenylphosphinocopper(I) hydride and the crystal structure of $H_6Cu_6(PPh_3)_6{\cdot}HCONMe_2$. *Inorg Chem* 1972; **11**: 1818-1825.

S5. Han H, Yao Y, Bhargava A *et al*. Tertiary hierarchical complexity in assemblies of sulfur-bridged metal chiral clusters. *J Am Chem Soc* 2020; **142**: 14495-14503.

S6. Hu W, Liu H, Zhang Y *et al*. Methanol steam reforming for hydrogen production driven by an atomically precise Cu catalyst. *Green Energy Environ* 2024; **9**: 1079-1084.

S7. Butt AM, Nag A, Managutti PB *et al*. Method-induced isomerism and concentration mediated isolation of two (Cu_{14} and Cu_{41}) atomically precise copper nanoclusters. *Chem Methods* 2025; **5**: e202400031.

S8. Nematulloev S, Huang R-W, Yin J *et al*. $[Cu_{15}(PPh_3)_6(PET)_{13}]^{2+}$: a copper nanocluster with crystallization enhanced photoluminescence. *Small* 2021; **17**: 2006839.

S9. Zhuo H-Y, Su H-F, Cao Z-Z *et al*. High-nuclear organometallic copper(I)-alkynide clusters: thermochromic near-infrared luminescence and solution stability. *Chem Eur J* 2016; **22**: 17619-17626.

S10. Sun C, Mammen N, Kaappa S *et al*. Atomically precise, thiolated copper-hydride nanoclusters as single-site hydrogenation catalysts for ketones in mild conditions. *ACS Nano* 2019; **13**: 5975-5986.

S11. Chen A, Kang X, Jin S *et al*. Gram-scale preparation of stable hydride $M@Cu_{24}$ (M = Au/Cu) nanoclusters. *J Phys Chem Lett* 2019; **10**: 6124-6128.

S12. Tang S, Song T, Cai X *et al*. Nitrate electroreduction to ammonia catalysed by atomically precise $Au_{28}Cu_{12}$ clusters. *Chem Commun* 2024; **60**: 7785-7788.

S13. Dong C, Huang R-W, Chen C *et al*. $[Cu_{36}H_{10}(PET)_{24}(PPh_3)_6Cl_2]$ reveals surface vacancy defects in ligand-stabilized metal nanoclusters. *J Am Chem Soc* 2021; 143: 11026-11035.

S14. Yuan P, Chen R, Zhang X *et al*. Ether-soluble Cu_{53} nanoclusters as an effective precursor of high-quality CuI films for optoelectronic applications. *Angew Chem Int Ed* 2019; **58**: 835-839.

S15. Kresse G, Furthmüller J. Efficient iterative schemes for ab initio total-energy calculations using a plane-wave basis set. *Phys Rev B* 1996; **54**: 11169-11186.

S16. Kresse G, Furthmüller J. Efficiency of ab-initio total energy calculations for metals and semiconductors using a plane-wave basis set. *Comput Mater Sci* 1996; **6**: 15-50.

S17. Perdew JP, Burke K, Ernzerhof M. Generalized gradient approximation made simple. *Phys Rev Lett* 1996; **77**: 3865-3868.

S18. Blöchl PE. Projector augmented-wave method. *Phys Rev B* 1994; **50**: 17953-17979.

S19. Grimme S. Semiempirical GGA-type density functional constructed with a long-range dispersion correction. *J Comput Chem* 2006; **27**: 1787-1799.

S20. Mi Z, Li Y, Wu C *et al.* CoO_x clusters-decorated IrO_2 electrocatalyst activates NO_3^- mediator for benzylic C-H activation. *Nat Commun* 2025; **16**: 3424.

S21. Seo B, Lee WH, Sa YJ *et al.* Electrochemical oxidation of toluene with controlled selectivity: The effect of carbon anode. *Appl Surf Sci* 2020; **534**: 147517.

S22. Yin Z, Gao Z, Luo L *et al.* A green and efficient electrocatalytic route for the highly-selective oxidation of C-H bonds in aromatics over 1D Co_3O_4-based nanoarrays. *Angew Chem Int Ed* 2025; **64**: e202415044.

S23. Nikl J, Hofman K, Mossazghi S *et al.* Electrochemical oxo-functionalization of cyclic alkanes and alkenes using nitrate and oxygen. *Nat Commun* 2023; **14**: 4565.

S24. Lv Y, Kong A, Zhang H *et al.* Electrocatalytic oxidation of toluene into benzaldehyde based on molecular oxygen activation over oxygen vacancy of heteropoly acid. *Appl Surf Sci* 2022; **599**: 153916.

S25. Zhu Y, Wang H, Jin K *et al.* Nanopolyaniline coupled with an anticorrosive graphene as a 3D film electrocatalyst for efficient oxidation of toluene methyl C-H bonds and hydrogen production at low voltage. *Chem Eur J* 2019; **25**: 6963-6972.

S26. Sarma BB, Efremenko I, Neumann R. Oxygenation of methylarenes to benzaldehyde derivatives by a polyoxometalate mediated electron transfer-oxygen transfer reaction in aqueous sulfuric acid. *J Am Chem Soc* 2015; **137**: 5916-5922.

S27. Shen Y, Yan Z, Wang K. Cobalt(II) mediated electro-oxidation of toluene and its derivatives. *Chem Eng J* 2024; **488**: 150857.

S28. Luo L, Zhu Y-Q, Chen W *et al.* Photoelectrocatalytic activation of C-H bond in toluene by titanium dioxide-supported subnanometric PtO_x clusters. *Angew Chem Int Ed* 2025; **64**: e202505544.
